# The structural, magnetic and optical properties of TM_n_@(ZnO)_42_ (TM = Fe, Co and Ni) hetero-nanostructure

**DOI:** 10.1038/s41598-017-16532-w

**Published:** 2017-11-28

**Authors:** Yaowen Hu, Chuting Ji, Xiaoxu Wang, Jinrong Huo, Qing Liu, Yipu Song

**Affiliations:** 1 Department of Physics, Tsinghua University, Beijing, 100084 China; 2 Department of Physics, University of Science and Technology Beijing, Beijing, 100083 China; 3 Department of Cloud Platform, Beijing Computing Center, Beijing, 100094 China; 4 Center for Quantum Information, IIIS, Tsinghua University, Beijing, 100084 China

## Abstract

The magnetic transition-metal (TM) @ oxide nanoparticles have been of great interest due to their wide range of applications, from medical sensors in magnetic resonance imaging to photo-catalysis. Although several studies on small clusters of TM@oxide have been reported, the understanding of the physical electronic properties of TM_n_@(ZnO)_42_ is far from sufficient. In this work, the electronic, magnetic and optical properties of TM_n_@(ZnO)_42_ (TM = Fe, Co and Ni) hetero-nanostructure are investigated using the density functional theory (DFT). It has been found that the core-shell nanostructure Fe_13_@(ZnO)_42_, Co_15_@(ZnO)_42_ and Ni_15_@(ZnO)_42_ are the most stable structures. Moreover, it is also predicted that the variation of the magnetic moment and magnetism of Fe, Co and Ni in TM_n_@ZnO_42_ hetero-nanostructure mainly stems from effective hybridization between core TM-3d orbitals and shell O-2p orbitals, and a magnetic moment inversion for Fe_15_@(ZnO)_42_ is investigated. Finally, optical properties studied by calculations show a red shift phenomenon in the absorption spectrum compared with the case of (ZnO)_48_.

## Introduction

Semiconducting hybrid materials with improved functionalities such as optical, electric and magnetic properties have been considered as potential candidates for a wide range of applications. For example, Rh, Pd and Pt particles supported on oxides, such as CeO_2_ and Al_2_O_3_, are widely used in catalysis^1,2^; magnetic iron-oxide nanoparticles have been investigated as contrast agents for magnetic resonance imaging^3^, which is of important use in cancer therapy. In particular, the physical properties of ZnO doped with ions of transition metal elements have been one of the most intriguing research topics in current materials science^4–9^. The characteristics of ZnO with Zn being a transition metal enables it to easily dope magnetic transition metal (TM) ions such as Mn^2+^, Fe^3+^, Co^2+^ and Ni^2+^ in place of Zn^2+^ in the crystal of ZnO. Dietl *et al*. discovered room temperature ferromagnetism in Mn- doped ZnO thin film, receiving tremendous attention to ZnO based materials^10^. Since then several studies have been carried out in ZnO based materials with different combinations of TM ions^4,11,12^. Some reports revealed the importance of point defects such as oxygen, zinc vacancies and interstitials in magnetic ordering^13,14^. Mishra and Das^15^ studied the optical characteristics of Fe-doped ZnO nanoparticles using FTIR. Sawalha *et al*.^16^ investigated the electrical conductivity of pure and doped ZnO ceramic systems. Their experiments indicated that donor concentration, point defects, and adsorption–desertion of oxygen were affected by the Fe doping for ZnO. Moreover, Shi and Duan^17^ studied the magnetic properties of TM (Cr, Fe and Ni) doped in ZnO nanowires by first-principles theory. Xiao *et al*.^18^ calculated the structural and electronic properties of Fe-doped ZnO nanoparticles, and the results showed that Fe doped ZnO nanoparticles were structurally more stable than the isolated FeO and ZnO phases.

In recent years, core-shell structures in which metals form the core and ZnO constitutes the shell have attracted intense interest due to their significantly high effectiveness in improving the photo-catalytic activity and the synergistic effect among components^19–22^. The core-shell architecture avoids exposing the inner core to the environment and thus maximizes the interaction between the building blocks. Moreover, the composition, size and morphology of the inner core and outer shell are important aspects of structural property and would most probably affect its stability. So far, to the best of our knowledge, investigations on the physical mechanism for the effect of composition, size and morphology of magnetic TM-core**/**ZnO-shell heterogeneous nanoparticles are very rare. Here, we report the theoretical studies on a series of TM_n_@(ZnO)_42_ (TM = Fe, Co and Ni) heterostructures by using the density functional theory (DFT). The structural, magnetic and optical properties of such core-shell heterostructures have been investigated. Variation of magnetic moment are studied and stable structures are founded among different models, especially for the moment inversion of Fe_15_@(ZnO)_42_. Furthermore, a red shift phenomenon is also obtained for the absorption spectrum of Fe_15_@(ZnO)_42_ compared with the case of (ZnO)_48_. We expect that our results for TM_n_@(ZnO)_42_ can help to understand the effects of the encapsulation on the structure, stability, and magnetic properties of TM clusters.

## Results and Discussion

### The structural properties of TM_n_@(ZnO)_42_ hetero-nanostructure

In simulation, due to the multiplicity and indeterminacy of core-shell hetero-structure, it is always a challenge to optimize the stable structure of metal-oxide heterogeneous with increasing number of atoms. In the following calculations, the TM_n_@(ZnO)_42_ core-shell model is built to investigate stable structure of TM_n_@(ZnO)_42_ with different n (n = 6–18). Considering the rationality of the structure, the magic number nanostructure of (ZnO)_48_ with *D*_*3d*_ symmetry is firstly chosen to be the initial configurations due to the fact that the (ZnO)_48_ model has the highest binding energy^23^. Therefore, six ZnO in the center of relaxed (ZnO)_48_ are removed, and magnetic TM-core TM_n_ clusters are constructed. The central empty position to put the magnetic TM-core relies on their lowest energy configurations according to the literatures^24^ with some considerations on the chemical bond length and interatomic interaction. Then, core-shell nanostructures of TM_n_@(ZnO)_42_ which contain TM_n_ inner core atoms and ZnO outer shell with 42 pairs of Zn-O atoms are built. First, all atoms are fully relaxed by using conjugate gradient algorithm and reach the criteria of the convergence tolerance for energy and maximum force. To further test the thermodynamic stability, we perform first-principles molecular dynamic simulations with a Nose-Hoover thermostat at 500 K in the canonical *NVT* ensemble. During the whole process of 10 ps simulations, the trajectories are calculated with a chosen time step of 1 fs. We find that there is no structure transform of the Fe_n_@(ZnO)_42_ core-shell phase, except for Fe_7_@(ZnO)_42_ (see the Supporting Information I). Then, we optimize the structure of the annealed Fe_7_@(ZnO)_42_ and replace the original structure. These results suggest that combination of the structure optimization and molecular dynamic simulations is needed for the precise prediction of structure.

**Figure 1:**
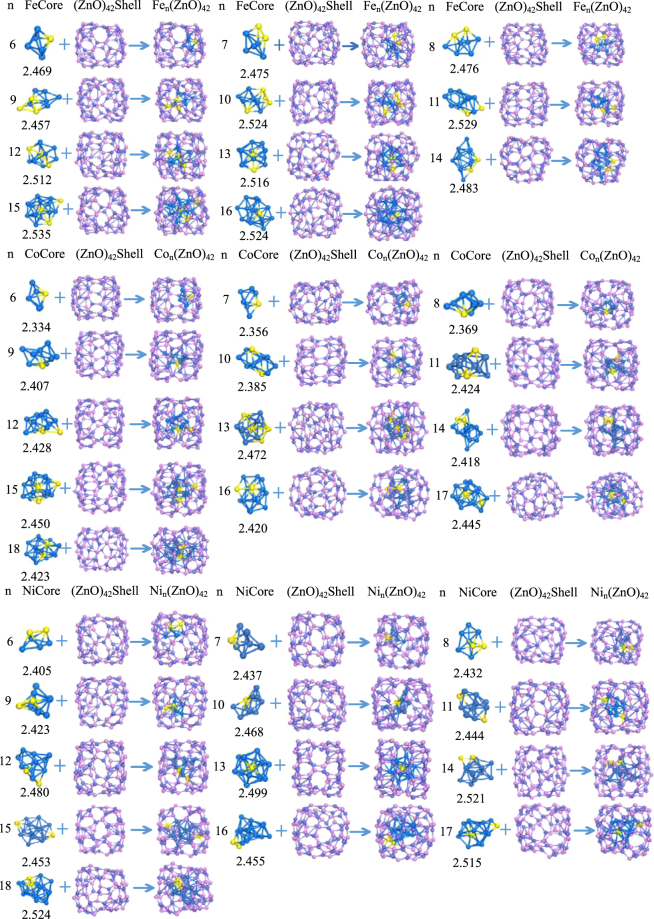
The optimized geometries of TM_n_@(ZnO)_42_ core-shell nanostructure. The pink, purple and blue balls show the positions of O, Zn and TM atoms, respectively. The small or abnormal magnetic moment of TM atoms are shown by yellow balls. The numbers below the inner core configurations indicate the average bond lengths (Å) within 3.00 Å (see Supporting Information III and supporting information I for enlarged picture of core-shell structures).

**Figure 2:**
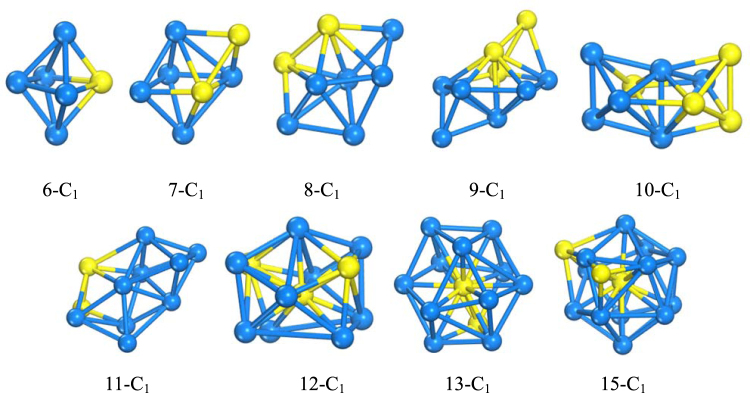
The optimized geometries of Fe inner-core of each size (n = 6–13 and 15).

According to our scheme, the stable configurations of TM_n_@(ZnO)_42_ clusters are obtained as shown in Fig. 1. The inner core TM_n_ and outer shell (ZnO)_42_ configurations separated from the optimized geometry configurations of TM_n_@(ZnO)_42_ are also illustrated in Fig. 1. It is noted that the encapsulated TM_n_ (n = 6–7 for Fe, n = 6–9 for Co and Ni) clusters shift towards the (ZnO)_42_ inside surface, indicating the presence of an attractive interaction of the TM_n_ clusters caused by the (ZnO)_42_ inside surface. However, large TM_n_ clusters (n = 8–16 for Fe, n = 10–18 for Co and Ni) are nearly located at the center of the cages due to the inner core TM_n_ cluster and outer shell cage sizes. The shells of n from 6 to 12 are a cage-like structure while the shells of n ≥ 13 have a tendency to change into a sphere, which may imply that with the increase of n, the shell is increasingly inclined to become a spherical structure. The exact symmetry for each TM cluster is C_1_ except that Ni_12_ is C_2_. The nine kinds of Fe_n_ (n = 6–13 and 15) inner core configurations picked from the optimized geometry configurations of Fe_n_@(ZnO)_42_ are displayed in Fig. 2 for the convenience of comparison with the structure of Fe_n_ clusters demonstrated in the literature^24^. It can be seen that large parts of the core structure of Fe_n_@(ZnO)_42_ are not similar to the case of Fe_n_, which is mainly due to the TM-oxygen interaction. Furthermore, it is intriguing that, in the TM_n_ clusters, the TM atom located at the prominent position and the center of TM_n_ (yellow balls in Fig. 1) have relatively small local magnetic moments. Therefore, there is a strong tendency of the magnetic TM_n_ clusters for lower symmetry structures, which helps to increase their energy stability due to the splitting of the highest occupied states. From the results of bond lengths (see Fig. 1), the Fe_n_ clusters are much more non-compact than the Co_n_ and Ni_n_ structures, indicating that the core is more close to shell for Fe_n_@(ZnO)_42_. This trend may affect the magnetic moments (see Supporting Information II) of the TM_n_@(ZnO)_42_ systems and induce more abnormal effect. (e.g. the atom with larger local magnetic moments for Fe shows inversion for the Fe atoms close to O atoms).

**Figure 3:**
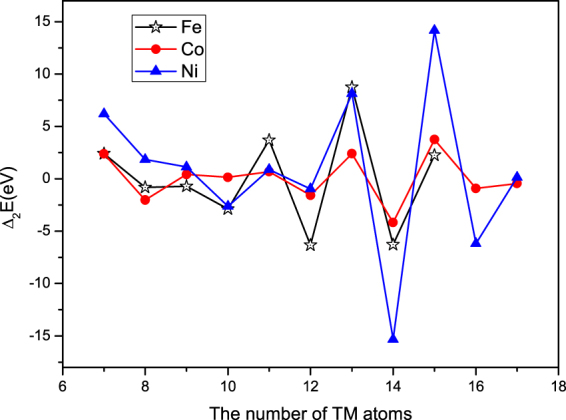
The second-order differences of total energies *Δ*_2_ *E*_*n*_ of TM_n_@(ZnO)_42_ nanostructure. It is noted that the largest *Δ*_2_ *E*_*n*_ are found at n = 13, 15 and 15 for TM = Fe, Co and Ni in TM_n_@(ZnO)_42_ core-shell structures, respectively, indicating that Fe_13_@(ZnO)_42_, Co_15_@(ZnO)_42_ and Ni_15_@(ZnO)_42_ are the most stable structure.

To investigate the structural stability, second-order differences of total energies (*Δ*_2_ *E*) for TM_n_@(ZnO)_42_ nanostructure are calculated and displayed in Fig. 3. The second-order differences of total energies are calculated by equation:

$${\Delta }_{2}{E}_{n}={E}_{n-1}+{E}_{n+1}-2{E}_{n}$$

where *E*_*n*_ and *n* refer to the total energy of TM_n_@(ZnO)_42_ and the number of TM atoms, respectively.

As shown in Fig. 3, the relatively large peaks of *Δ*_2_ *E*_*n*_ are found at n = 13, 15 and 15 for TM = Fe, Co and Ni in TM_n_@(ZnO)_42_ core-shell structures, respectively, demonstrating that Fe_13_@(ZnO)_42_, Co_15_@(ZnO)_42_ and Ni_15_@(ZnO)_42_ are the most stable configurations among all the clusters in the size range of the present study. It is also find that clusters of 7, 13 and 15 atoms are particularly stable, and these three sizes for metal cluster are well-known “magic numbers”^25^. The calculated Zn-O bond lengths and O-Zn-O bond angle of the (ZnO)_48_ and M@ZnO (we use M@ZnO to represent all TM_n_@(ZnO)_42_ for TM = Fe, Co, Ni and n = 13, 15, 15 in the following discussion) are listed in Table 1 together with other calculated work^23^, from which it can be seen that our results of (ZnO)_48_ reach an agreement with the other studies^23^. It is also obvious that there is a contraction behavior for the outer-shell of M@ZnO compared with (ZnO)_48_, indicating that doping at the center with a magnetic TM atom could provide strong bonding among surface atoms, that is, the Zn-O bonding of M@ZnO is stronger than the (ZnO)_48_ cluster due to the interaction of M-O.

**Table 1:** The bond length and bond angle of both the (ZnO)_48_ and M@ZnO core-shell nanostructures, and the values in parenthesis are from the calculations of literature^29^.

	(ZnO)_48_	Fe_13_@(ZnO)_42_	Co_15_@(ZnO)_42_	Ni_15_@(ZnO)_42_
Zn-O bond length (Å)	1.875–2.201 (1.842–2.206)	1.869–2.137	1.852–2.177	1.859–2.184
O-Zn-O bond angle (°)	92.3–174.3 (90.2–172.2)	89.612–161.336	88.257–157.0524	88.857–157.152

### The magnetic and electronic structure properties of TM_n_@(ZnO)_42_ hetero-nanostructure

The magnetic properties of encapsulated TM_n_ (TM = Fe, Co and Ni) clusters inside (ZnO)_42_ are calculated based on the stable geometries discussed above. All of the transition metal atom magnetic moments of the TM_n_@(ZnO)_42_ core-shell nanostructure are shown in Tables 2–4. More details of magnetic moments are described in the Supporting Information II. The following trends can be observed: (i) Except for a few cases, the magnetic moments decrease from outside to inside for core transition metal atoms. For example, for relatively stable structure Fe_15_@(ZnO)_42_, Co_15_@(ZnO)_42_, and Ni_13_@(ZnO)_42_, the center Fe, Co, Ni atoms have the magnetic moments 1.996, 1.167, and 0.226 μ_B_/atom, which are significantly smaller than the other magnetic moments such as 2.64, 1.78, and 0.68 μ_B_/atom, the average value for Fe, Co and Ni, respectively. (ii) As is presented in Table 5, a general feature is that local magnetic moments tend to have some relationship with the TM-O distance and the small distance corresponds to a small magnetic moment. Especially for several Fe_n_@(ZnO)_42_ systems, e.g., Fe_15_@(ZnO)_42_, it is found that some Fe local magnetic solutions change from ferromagnetic to antiferromagnetic phases (e.g. −2.176 μ_B_/atom) with the Fe-O distance decreased. A similar phenomenon can also be found in the TM@Mg_12_O_12_^26^ and TM_*m*_@C_*n*_^27^. (iii) For most of the systems, we observed a large number of atomic configurations with slightly different magnetic moments. These results indicate that one of the magnetic configurations might be more favorable or a wide range of magnetic configurations might exist at real experimental conditions, and experimental techniques might access only the average results.

**Table 2:** Atoms magnetic moments (μ_B_) for Fe_n_@ZnO_42_ (LDA).

	n = 6	n = 7	n = 8	n = 9	n = 10	n = 11	n = 12	n = 13	n = 14	n = 15	n = 16
Fe1	2.800	−**0.820**	2.717	2.745	2.919	2.713	2.850	2.758	2.897	2.676	2.668
Fe2	2.772	2.622	2.763	2.532	2.791	2.542	2.559	2.619	2.712	2.603	2.317
Fe3	2.631	2.800	2.717	2.832	−**0.336**	2.626	1.535	2.748	2.695	2.710	2.638
Fe4	2.748	2.283	2.778	2.707	−**2.554**	2.734	−**2.506**	2.815	2.707	2.619	2.841
Fe5	2.284	2.399	2.560	2.826	2.724	2.694	2.750	2.563	**−1.027**	1.996	1.767
Fe6	2.529	2.601	2.536	2.718	−**0.636**	2.731	2.862	2.813	2.814	2.616	2.458
Fe7		2.676	2.674	2.599	0.335	2.678	2.898	1.537	2.863	2.772	2.355
Fe8			2.827	2.789	2.149	2.653	2.770	2.665	2.761	2.332	2.366
Fe9				−0.023	2.468	2.782	2.652	2.786	2.599	**−2.176**	2.508
Fe10					2.780	2.545	2.801	2.459	2.800	2.778	2.996
Fe11						2.693	2.494	2.770	2.637	2.784	2.807
Fe12							−**2.522**	2.816	2.473	2.862	2.514
Fe13								2.544	2.789	2.340	2.571
Fe14									2.831	−**0.767**	2.568
Fe15										2.577	2.699
Fe16											2.742

**Table 3:** Atoms magnetic moments (μ_B_) for Fe_n_@ZnO_42_ (LDA + U).

	n = 6	n = 7	n = 8	n = 9	n = 10	n = 11	n = 12	n = 13	n = 14	n = 15	n = 16
Fe1	2.961	3.219	3.209	3.089	3.169	3.020	3.219	3.103	3.140	3.143	3.103
Fe2	2.967	3.264	3.088	2.810	3.191	3.051	2.902	3.049	3.131	2.884	2.896
Fe3	3.083	3.100	3.140	3.244	3.201	3.070	2.272	3.067	1.030	2.976	3.047
Fe4	3.105	3.065	3.115	3.195	2.987	3.019	3.101	3.051	3.025	3.020	3.220
Fe5	2.805	3.130	3.133	3.075	3.041	3.131	3.156	3.062	3.239	2.718	2.565
Fe6	3.200	3.218	3.224	3.174	3.188	3.169	3.166	3.093	3.140	3.079	3.118
Fe7		3.164	3.047	3.027	3.137	3.155	3.193	2.352	3.266	3.194	3.049
Fe8			2.986	3.165	2.844	2.866	3.191	3.107	3.069	3.073	2.857
Fe9				3.187	3.016	3.142	3.055	3.188	3.120	3.093	2.994
Fe10					3.245	3.075	3.183	2.967	3.208	3.174	3.389
Fe11						2.993	3.030	3.093	3.204	3.058	3.035
Fe12							3.164	3.160	2.849	3.221	3.074
Fe13								3.021	3.199	2.996	3.061
Fe14									3.062	3.093	3.081
Fe15										3.123	3.115
Fe16											3.173

**Table 4:** Atoms magnetic moments (μ_B_) for TM_n_@ZnO_42_ (M = Co,Ni).

	Co_n_@ZnO_42_				Ni_n_@ZnO_42_					
	n = 12	n = 13	n = 14	n = 15	n = 8	n = 13	n = 14	n = 15	n = 16	n = 17
M1	1.631	1.879	1.808	1.861	0.454	0.750	0.527	0.563	0.541	0.612
M2	1.737	1.811	1.816	1.704	**0.277**	0.742	0.635	0.507	0.618	0.532
M3	1.768	**0.638**	1.991	1.852	0.561	**0.226**	0.690	0.713	0.537	**0.307**
M4	1.797	1.772	1.802	1.745	0.376	0.664	0.536	0.439	0.529	0.655
M5	1.833	1.803	1.825	1.790	0.381	0.663	0.609	0.511	**0.281**	0.683
M6	1.801	1.800	1.889	1.792	0.485	0.649	0.593	0.511	0.582	0.677
M7	1.804	1.753	1.805	1.700	0.566	0.664	**0.498**	**0.381**	0.638	0.624
M8	1.722	1.803	1.899	**1.167**	**0.205**	0.651	0.639	0.557	0.482	0.522
M9	1.746	1.802	1.790	1.802		0.663	**0.458**	0.580	0.504	0.548
M10	1.674	1.770	1.963	1.816		0.655	0.730	0.574	0.455	**0.280**
M11	1.658	1.810	1.792	1.701		0.741	0.620	0.517	0.652	0.544
M12	1.830	1.753	1.828	1.761		0.655	0.606	0.618	**0.380**	0.543
M13		1.878	1.843	1.798		0.750	0.705	**0.423**	0.703	0.391
M14			1.859	1.785			0.523	0.594	0.535	0.495
M15				1.800				0.623	0.577	0.318
M16									0.678	0.385
M17										0.537

**Table 5:** TM-O distance for TM_n_@ZnO_42_ (TM = Fe,Co,Ni).

	Fe_15_@ZnO_42_		Co_15_@ZnO_42_		Ni_13_@ZnO_42_	
	magnetic moments (μB)	Fe-O distance	magnetic moments(μB)	Co-O distance	magnetic moments (μB)	Ni-O distance
M1	2.676	1.997	1.861	**4.021**	0.750	2.04
M2	2.603	**3.219**	1.704	1.969	0.742	1.975
M3	2.710	**3.086**	1.852	1.916	**0.226**	–
M4	2.619	1.915	1.745	1.945	0.664	**3.364**
M5	1.996	—	1.790	2.009	0.663	1.964
M6	2.616	1.975	1.792	1.973	0.649	1.955
M7	2.772	1.986	1.700	1.922	0.664	1.963
M8	2.332	2.91	**1.167**	—	0.651	1.955
M9	−**2.176**	1.818	1.802	1.99	0.663	**3.824**
M10	2.778	1.973	1.816	**3.670**	0.655	2.14
M11	2.784	**3.181**	1.701	**3.442**	0.741	1.974
M12	2.862	1.995	1.761	1.936	0.655	2.139
M13	2.340	**3.650**	1.798	**3.327**	0.750	2.039
M14	−**0.767**	1.845	1.785	2.002		
M15	2.577	1.965	1.800	1.961		

**Figure 4:**
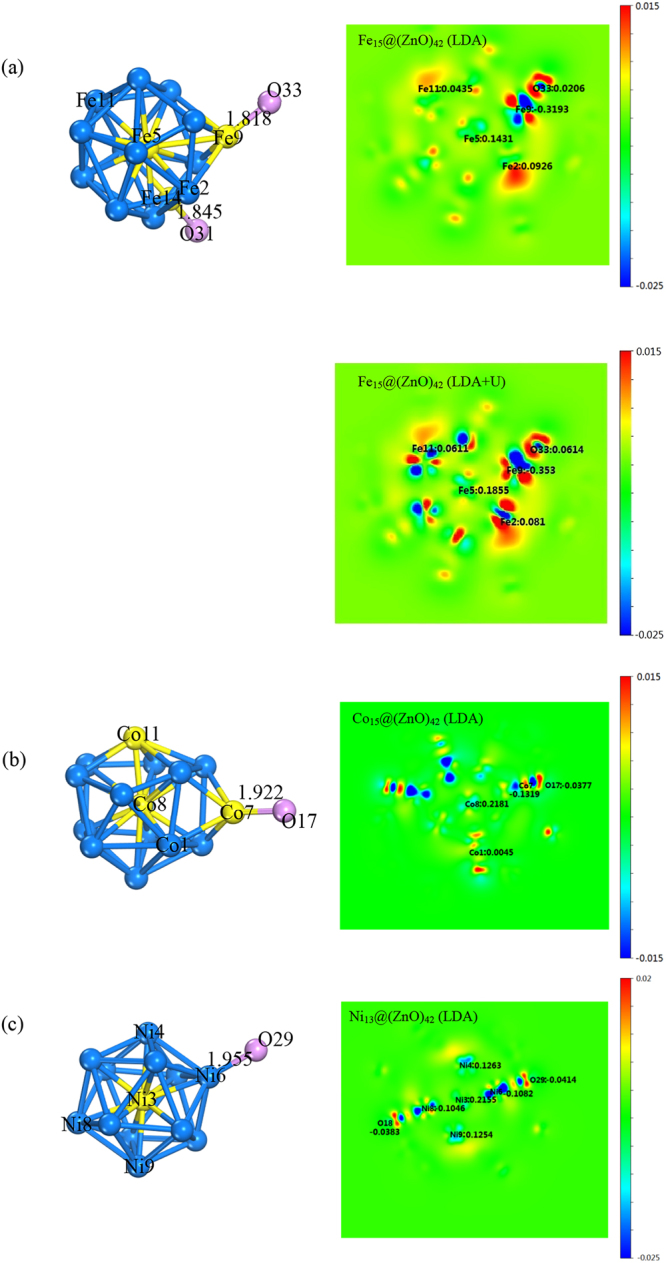
Plot of the 2D electron density difference and geometry configuration for (**a**) Fe_15_@(ZnO)_42_; (**b**) Co_15_@(ZnO)_42_ and (**c**) Ni_13_@(ZnO)_42_. The atom numbers which overlap with corresponding atom and the bond length (Å) are depicted on geometry configuration.

**Figure 5:**
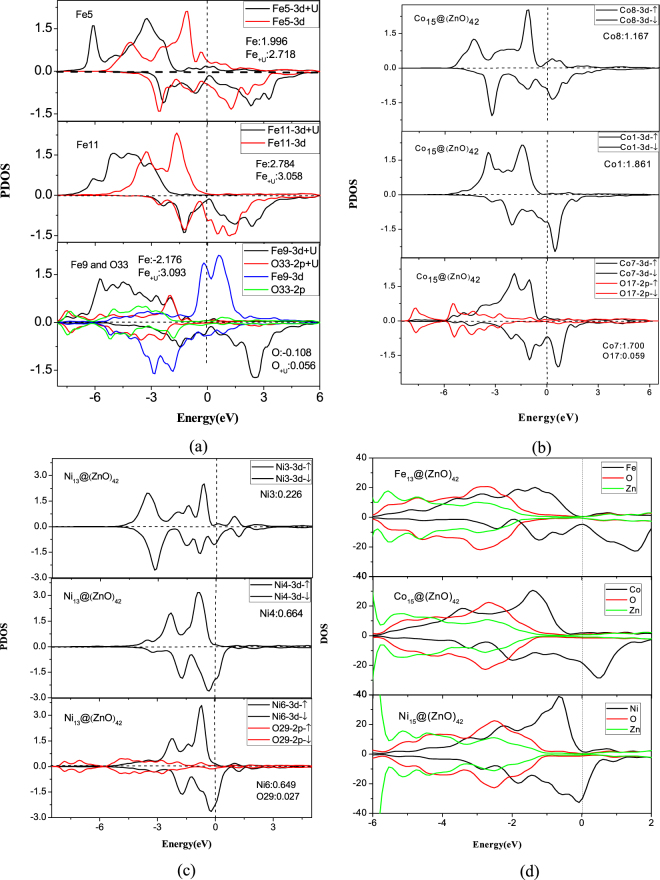
The partial DOS of (**a**) Fe_15_@(ZnO)_42_, (**b**) Co_15_@(ZnO)_42_ and (**c**) Ni_13_@(ZnO)_42_; and the total DOS of (**d**) M@ZnO; The dotted lines refer to the Fermi level. The unit is electrons/eV. The magnetic moment of some corresponding atom is also depicted inside each DOS and the unit is μB/atom.

To obtain a better understanding for the origin of TM magnetic moments difference, we take relatively stable compound mentioned above as examples to present the charge density difference and investigate the p-O and d-TM projected DOS (see Fig. 4 and Fig. 5). Charge transfer data of the typical atom have been marked out in Fig. 4, demonstrating that, apart from the transition metal atoms neighboring oxygen atoms, the charge transfer numbers increase from outside to inside for core transition metal atoms. For instance, the Fe, Co, Ni atoms at the center of core have the charge transfer numbers 0.1431, 0.2181 and 0.2155, which are much larger than the numbers of other transition metal atoms and correspond to smaller magnetic moments as discussed previously. More intriguingly, because of the interaction with O atoms, transition metal atoms near O atoms have a large charge transfer, leading to smaller magnetic moment and even magnetization reversal has been found.

Figure 5 shows the PDOS of the representative atoms for up (↑) and down (↓) spins, demonstrating differences in the shape of PDOS among transition metal atoms at different position. This shape differences are mainly a shift to high energy or low energy, which can be explained by the decrease or increase of the effective hybridization between core TM-3d orbitals and the shell O-2p orbitals, resulting in the charge transfer from core TM to shell O. The charge density difference is demonstrated in Fig. 4. Near to the Fermi level (${{\rm{E}}}_{{\rm{F}}}$), a large overlap between O 2p and TM 3d is clearly seen for atoms with minimal TM-O distance, showing a strong hybridization between O and TM atoms. This also explains why all the core-shell clusters have relative large core-shell interaction energy.

In the case of Fe_15_@(ZnO)_42_, as the interaction of TM-O increases, spin-split becomes significant for the d- projected DOS, resulting in a large magnetic moment change from the center of the core to the edge of the core. In Fig. 4, we take Fe5, Fe9 and Fe14 as an example. Fe5 is in the center of the core with a magnetic moment 1.996 μ_B_/atom. Fe9 and Fe14 are located on the edge of the core with a moment −2.714 μ_B_/atom and −0.767 μ_B_/atom, indicating an inverse direction compared with other moments. It can be seen that the O atoms neighboring Fe9 and Fe14 are O33 and O31 respectively. At the same time, these two O atoms have the largest two negative magnetic moments (−0.108 μ_B_/atom and −0.064 μ_B_/atom) and they have a very large charge transfer at the same time. So the moment inversion could be interpreted by the strong interaction between Fe-O atoms, which could also be utilized to understand the magnetic moment difference for different core atoms. Similar spin-split can also be seen in Co_15_@(ZnO)_42_ and Ni_13_@(ZnO)_42_, where the spin-up and spin-down DOS become asymmetric. For Co and Ni at more outer positions or with smaller TM-O distances, this asymmetry is more significant. For Co_15_@(ZnO)_42_ and Ni_13_@(ZnO)_42_, spin-split becomes less obvious because of the weaker TM-O interaction than Fe_15_@(ZnO)_42_, resulting in the less magnetic moment change. For instance, magnetic moment for Co_15_@(ZnO)_42_ is 1.167 μ_B_/atom for Co8 at the center of core, 1.861 μ_B_/atom (Co1) for atom at the edge of core but far from O atom. And 1.700 μ_B_/atom (Co7) for atom at the edge is close to O (O17) atom. At the same time, see Figs 4 and 5, the moment of O (O17) close to Co (Co7) is 0.059 μ_B_/atom and the moment of O (O29) near Ni (Ni6) is 0.027 μ_B_/atom, which is not large enough and leads to less TM moment changes. Moreover, in Fig. 4, less charge transfer from core Co or Ni to shell O also shows weaker interaction than Fe_15_@(ZnO)_42_.

In addition, it is indicated from Table 5 and Supporting Information II that the magnetic moment of transition metal is related to the coordination number, the average TM-TM bond length and the distance of TM-O, e.g. large coordination number usually lead to small magnetic moment; and a small bond length of TM-TM or TM-O also tends to result in a small moment. The variation moments of TM atom may arise from the contribution of the synergistic effect of the coordination number and bond length. For Co, Ni atoms at the center of the core, their moments are small due to the largest coordination number among all the core atoms. Although Co1 has a larger coordination number compared with Co11, the average bond length between Co1 and neighbor Co atoms (2.458 Å) is larger than the case of Co11 with adjacent Co atoms (2.448 Å). At the same time, the distance of Co-O atoms is largest for Co1-O. As a result, the final moment of Co1 is strongest in Co_15_@(ZnO)_42_, indicating that the magnetic moment of atoms are deeply related to the geometry configuration of each atoms, which is consistent with the result from charge transfer.

Indeed, although the 3*d* orbitals are usually spatially extended and the delocalization is even more enhanced by hybridization with oxygen orbitals in TM_n_@(ZnO)_42_, electron correlations can still play an important role in 3*d* systems, especially for Fe_n_@(ZnO)_42_ where the electronic configuration maximizes the correlation effects due to the delicate balance of charge states in Fe_n_@(ZnO)_42_. Thus, we then focus on the results obtained at Coulomb energy *U* = 4.5 eV and exchange parameter *J* = 0.89 eV for all Fe ions.

As given in Tables 2 and 3, a general feature is that inclusion of U leads to local magnetic moments increased. Comparing the results for the magnetic state with and without U, for example, for relatively stable structure Fe_15_@(ZnO)_42_, we find that U will lead to different correlation behaviors with various Fe-O distances: (I)The center Fe atom shows the magnetic moments 2.718 μ_B_/atom, which are smaller than the other values (3.056 μ_B_/atom), which is consistent with the LDA results. (II) For the medium Fe-O distance, Fe correspond to an intermediately correlated by U and magnetic moment tend to around 3 μ_B_/atom, which consistent with the literature value^28^. (III) Our LDA + *U* calculations finding a significant role of electron-electron correlations in small Fe-O distance atoms. Especially for the two nearest distances, it is found that the Fe local magnetic solutions change from antiferromagnetic (−2.176, −0.767) to ferromagnetic (3.093, 3.093) phases. From this point of view, stronger interaction of Fe-O makes the magnetic value and ordering more sensitive to correlation effects. And a similar phenomenon can also be found in the other Fe_n_@(ZnO)_42_ compounds.

This can also be observed by comparing the DOS between the results from LDA and LDA+U, given in the Fig. 5(a). In Fe_15_@(ZnO)_42_, as the interaction of TM-O increases, spin-split become more obvious, resulting in a large magnetic moment change as compared with LDA result. For Fe9, with the strong interaction between Fe-O atom, the DOS spin-up shifted to lower energy and spin-down pushed towards higher energy more significantly. And inclusion of U leads to a transition of the magnetic ordering, coinciding with the above analyses. At the same time, charge density difference also reflects the same tendency (see Fig. 4(a)).

### The optical properties of M@ZnO and (ZnO)_48_

**Figure 6:**
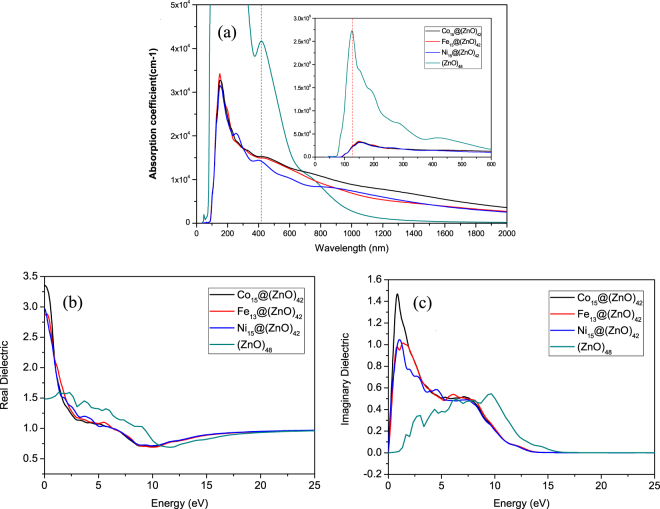
The calculated optical absorption (**a**), and dielectric function: real part (**b**) and imaginary part (**c**) of M@ZnO (M = Fe,Co,Ni) core-shell structure and (ZnO)_48_.

In order to investigate the influence of magnetic TM inner-core on the optical properties of ZnO shell cage, the dielectric function of M@ZnO and pure (ZnO)_48_ nanostructures are all calculated for comparison and the optical absorption of core-shell structure and pure (ZnO)_48_ is illustrated in Fig. 6. Compared with the (ZnO)_48_, the optical absorption peaks of core-shell structure Fe_13_@(ZnO)_42_, Co_15_@(ZnO)_42_ and Ni_15_@(ZnO)_42_ have an obvious red shift at 147.56 nm, compared with pure (ZnO)_48_ at 123.95 nm, which is due to the effect of Fe, Co, Ni core. The spectral line of the Ni_15_@(ZnO)_42_ appeared to have a smaller peak at about 255.84 nm while both Fe_13_@(ZnO)_42_ and Co_15_@(ZnO)_42_ have no peak there. According to the DOS of Ni_15_@(ZnO)_42_ (shown in Fig. 5(d)), we found that this smaller peak originates from the stronger interaction between Ni-O atoms and more abundant charge transfer of O–Zn atom (see Supporting Information II). We contrast the DOS of M@ZnO (Fig. 5(d)) and conclude that the influence on Zn-O interaction for the case of introducing Ni atoms is weaker than the case of Co, Fe atoms, particularly at <3 eV. Moreover, it is noted that, at around the 400 nm (in the visible light), all the core-shell structures and (ZnO)_48_ have a distinct peak, although with some differences in the height of the peak, coming from the contribution of electrons transfer of O–Zn atom at shell.

Figure 6 also exhibits the imaginary part and real part of dielectric function of M@ZnO and pure (ZnO)_48_. For the real part of dielectric function of Fe_13_@(ZnO)_42_, Co_15_@(ZnO)_42_ and Ni_15_@(ZnO)_42_, It is found that there are no obvious differences among them. Moreover, both of the real parts of dielectric functions of (ZnO)_48_ and M@ZnO are all positive. Unlike the M@ZnO, in the lower energy region (<3.2 eV), (ZnO)_48_ does not have a major peak and is quite smooth but in the higher energy region (>10 eV), all of the tendency of curves become consistent with each other. In addition, as the DOS presented in Fig. 5(d), M@ZnO shows a typical half-metallic behavior from spin majority and minority components, which is in keeping with the result of the real part of dielectric function. In addition, the spin polarization of Fe, Co and Ni is the major contribution for DOS around the Femi level.

Furthermore, the imaginary part of dielectric function shows that the curve of (ZnO)_48_ has no distinct peak while M@ZnO appears to have a larger peak at around 0.85–1.47 eV, which is mainly due to the contribution of Co, Fe, Ni atoms in the core. It indicates that there is an evident absorptive action in the infrared region and the margin of visible light, especially in the case of Fe_15_@(ZnO)_42_, whose peak of absorption is closer to the visible light region. Finally, due to the interaction between O atom in shell and metal atom in core, the peak at 9.68 eV of (ZnO)_48_ vanishes and the curve decreases to zero rapidly.

## Conclusions

The structural, magnetic and optical properties of TM_n_@(ZnO)_42_ (TM = Fe, Co and Ni) core-shell nanostructures are studied by the First-principles calculations. Our results indicate that Fe_13_@(ZnO)_42_, Co_15_@(ZnO)_42_ and Ni_15_@(ZnO)_42_ core-shell nanostructure are the most stable configurations. Compared with (ZnO)_48_ value, the Zn-O bonding of M@ZnO is stronger due to the interaction of TM-O. The special magnetism mainly effect by O atoms and TM atoms, which can be attributed to the strong TM-O hybridization and charge transfer. It is also found that this strong interaction induces some magnetic moment inversion for Fe_13_@(ZnO)_42_. Furthermore, the optical properties of M@ZnO are systematically investigated based on absorption coefficient. Compared with the absorption spectrum of the (ZnO)_48_, we find that an obvious red shift has occurred, and it is in accordance with the behavior of the calculated electronic structure.

## Methods

All calculations in this paper are performed in the VASP codes^29,30^ based on density functional theory (DFT)^31,32^ within the projector augmented wave (PAW)^33^. The exchange and correlation potential is treated with the generalized gradient approximation (GGA) methods as described by Perdew–Burke–Ernzerhof (PBE)^34^. The electron wave functions are expanded in plane wave with a cutoff energy of 480 eV. All atoms are fully relaxed and the convergence tolerance for energy and maximum force are set to 1.0 × 10^−5^ eV and −5 × 10^−3^ eV/Å. For k-point sampling, we use a single Γ point for the geometry optimizations in the first Brillouin zone. Spin-polarization is taken into account in this work. In a second step we supplement the LDA calculations by including a Coulomb energy *U* = 4.5 eV^35^ and exchange parameter *J* = 0.89 eV^36^ on Fe 3d orbitals within the LDA + U scheme. In the calculations, the free TM_n_@(ZnO)_42_ is located in a rectangular supercell with a size of 30 × 30 × 30 Å^3^. The interaction between periodic images could be neglected on this size.

In order to predict the stable structures, we perform ab initio molecular dynamics (AIMD) simulations. The initial configuration of the Fe_n_@(ZnO)_42_ is annealed at 500 K. MD simulations are carried out in the NVT ensemble with a time step of 1 fs for a total time of 10 ps. The temperature is controlled by using the Nosé–Hoover method^37^.

## Electronic supplementary material

Supporting Information
