# Supplementary material for: The structural, magnetic and optical properties of TMn@(ZnO)42 (TM = Fe, Co and Ni) hetero-nanostructure
Source: Sci Rep. 2017 Nov 28;7:16485. doi: 10.1038/s41598-017-16532-w (PMC5705660; doi:10.1038/s41598-017-16532-w)
Supplement: Supplementary file 1 — Supporting Information [file 41598_2017_16532_MOESM1_ESM.pdf]

Supporting Information

The structural, magnetic and optical properties of $TM_n@(ZnO)_{42}$ (TM = Fe, Co and Ni) hetero-nanostructure

Yaowen Hu[a], Chuting Ji[a], Xiaoxu Wang[b, c, †], Jinrong Huo[b, †], Qing Liu[b], and Yipu Song[d, *]
[a]Department of Physics, Tsinghua University, Beijing 100084, China
[b]Department of Physics, University of Science and Technology Beijing, Beijing 100083, China.
[c]Department of Cloud Platform, Beijing Computing Center, Beijing 100094, China
[d]Center for Quantum Information, IIIS, Tsinghua University, Beijing 100084, China

This file includes

Supporting Information I: Enlarged Picture of Core-Shell Structures
Supporting Information II: Electric Distribution and Coordination Number
Supporting Information III: Bond Length

Supporting Information I: Enlarged Picture of Core-shell Structures

The structural, magnetic and optical properties of $TM_n@(ZnO)_{42}$ (TM = Fe, Co and Ni) hetero-nanostructure

Yaowen Hu[a], Chuting Ji[a], Xiaoxu Wang[b, c, †], Jinrong Huo[b, †], Qing Liu[b], and Yipu Song[d, *]

[a]Department of Physics, Tsinghua University, Beijing 100084, China

[b]Department of Physics, University of Science and Technology Beijing, Beijing 100083, China.

[c]Department of Cloud Platform, Beijing Computing Center, Beijing 100094, China

[d]Center for Quantum Information, IIIS, Tsinghua University, Beijing 100084, China

This file gives an enlarged picture of each optimized $TM_n@(ZnO)_{42}$ core-shell structure. The pink, purple and blue balls show the positions of O, Zn and TM atoms, respectively. The small or abnormal magnetic moment of TM atoms are shown by yellow balls.

The left column gives the core-shell structure of $TM_n@(ZnO)_{42}$. The center column is the core of $TM_n@(ZnO)_{42}$ and the right column shows the shell of $TM_n@(ZnO)_{42}$.

1. $Fe_n@(ZnO)_{42}$

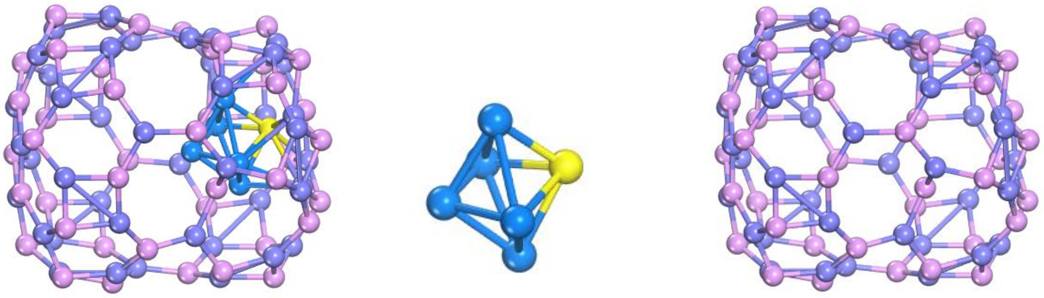

Fe6

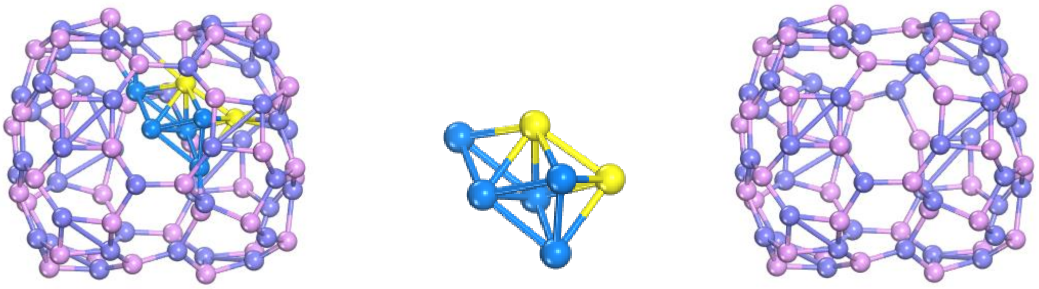

Fe7

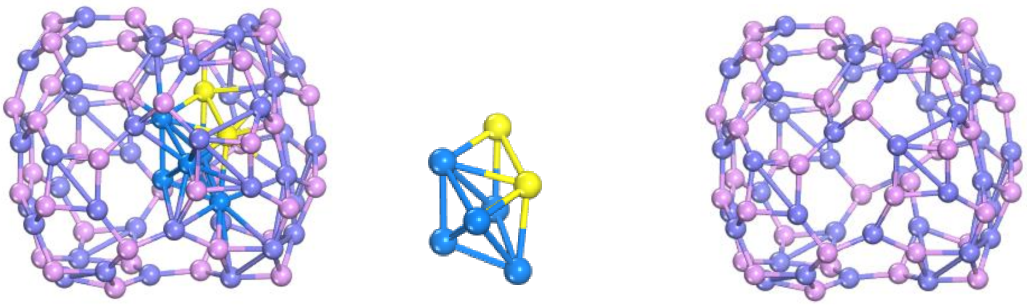

Fe7- the structure of the annealed + optimized $Fe_7@(ZnO)_{42}$

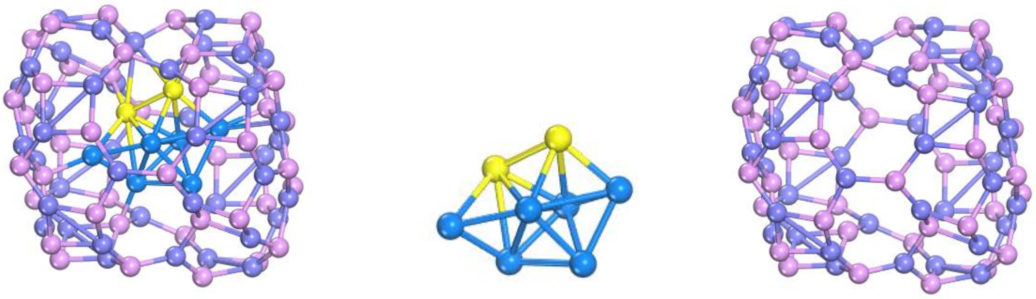

Fe8

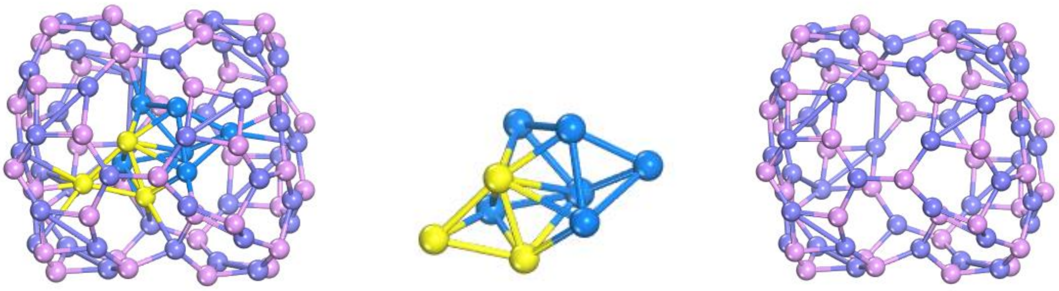

Fe9

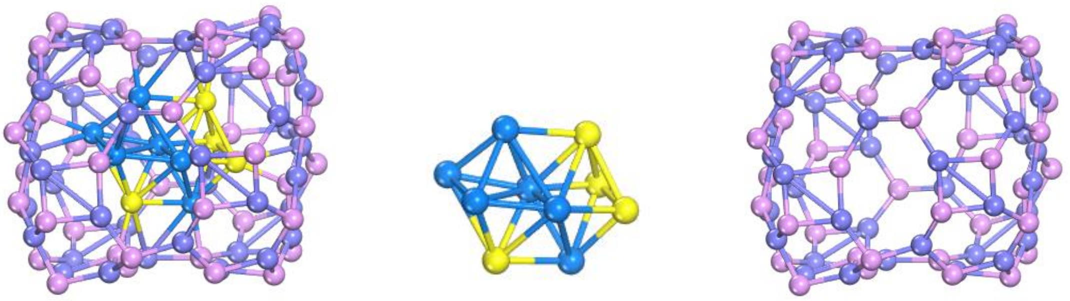

Fe10

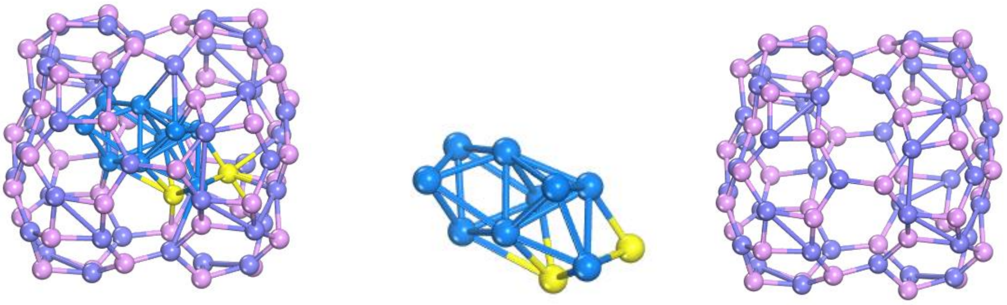

Fe11

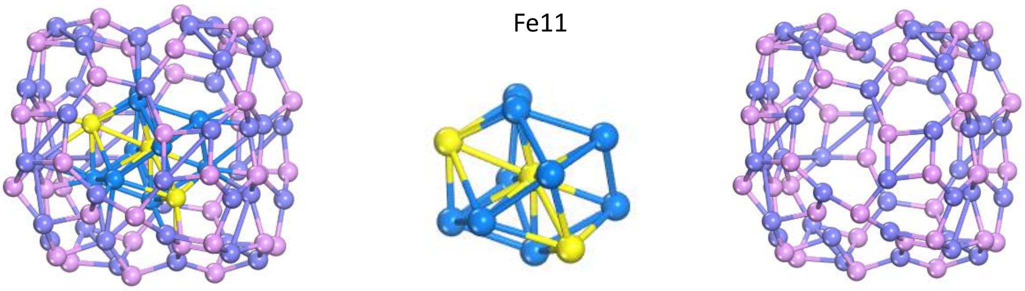

Fe12

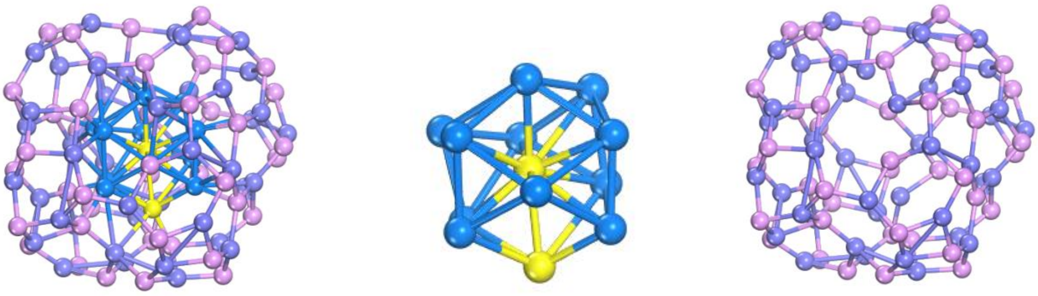

Fe13

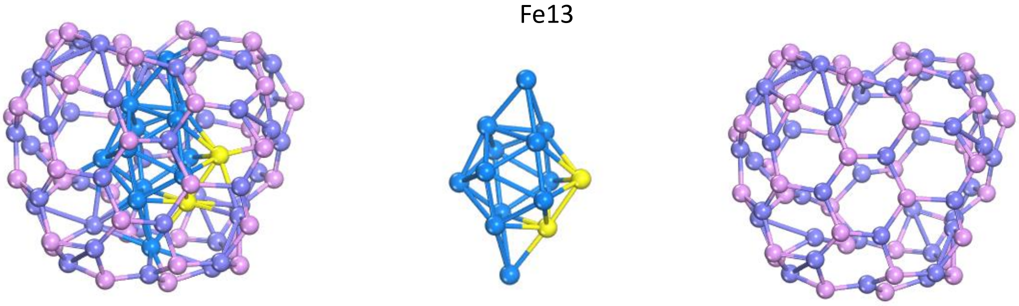

Fe14

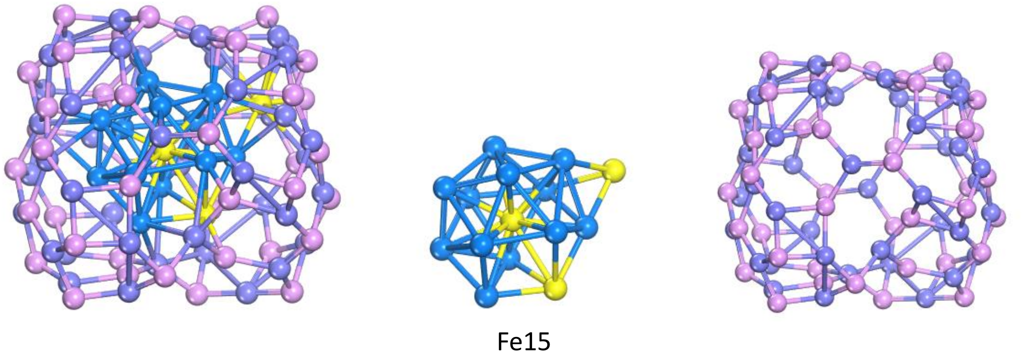

Fe15

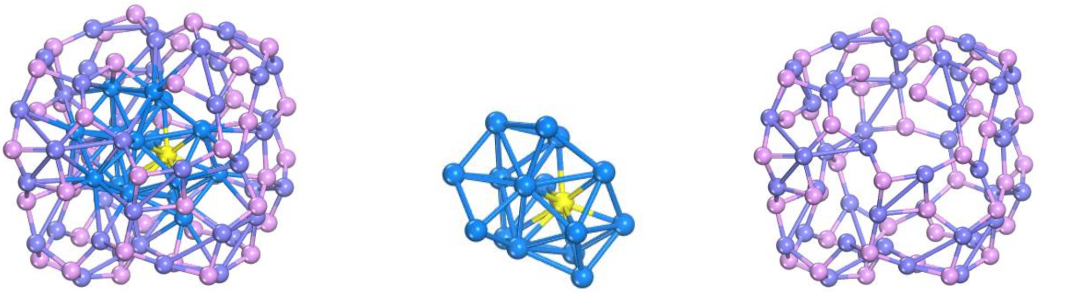

Fe16

2. $Co_n@(ZnO)_{42}$

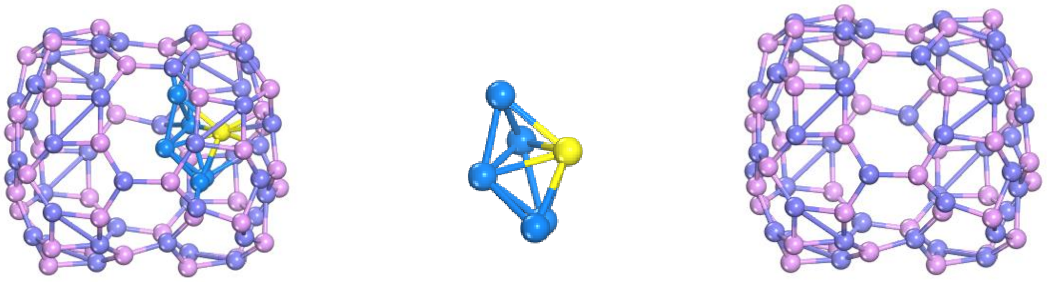

Co6

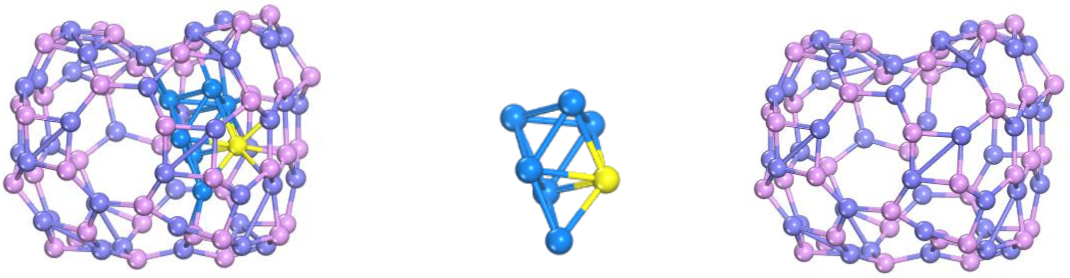

Co7

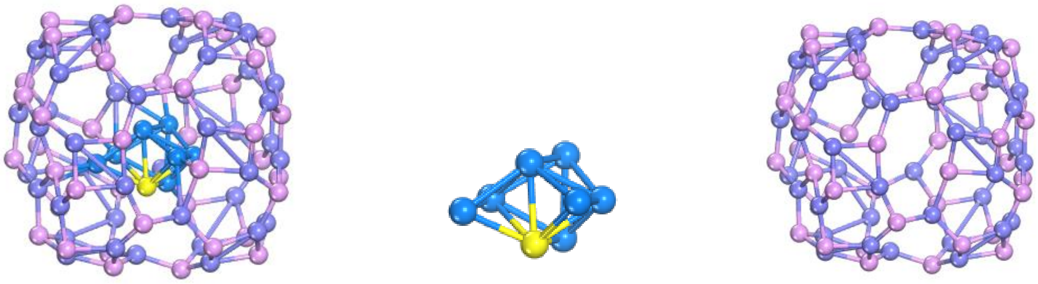

Co8

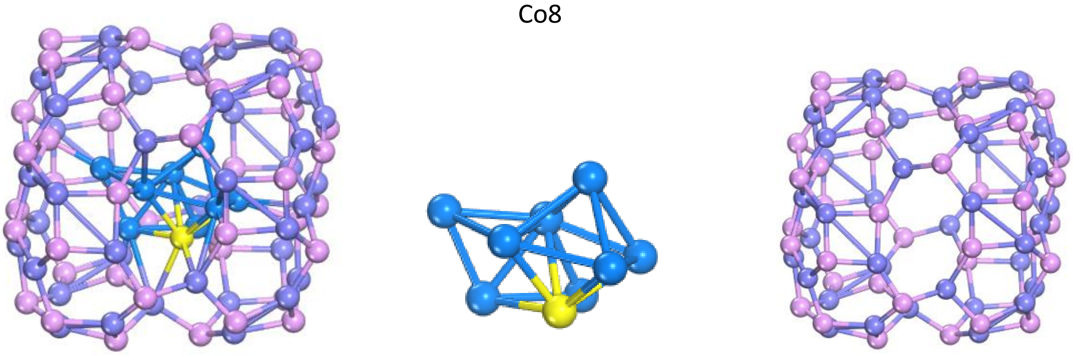

Co9

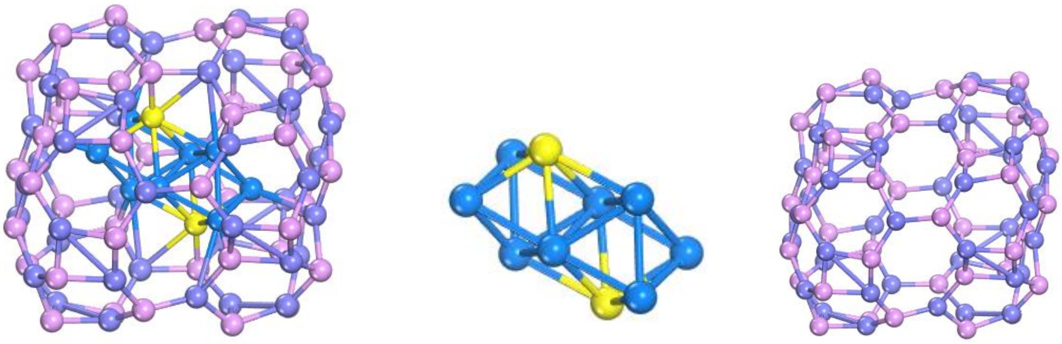

Co10

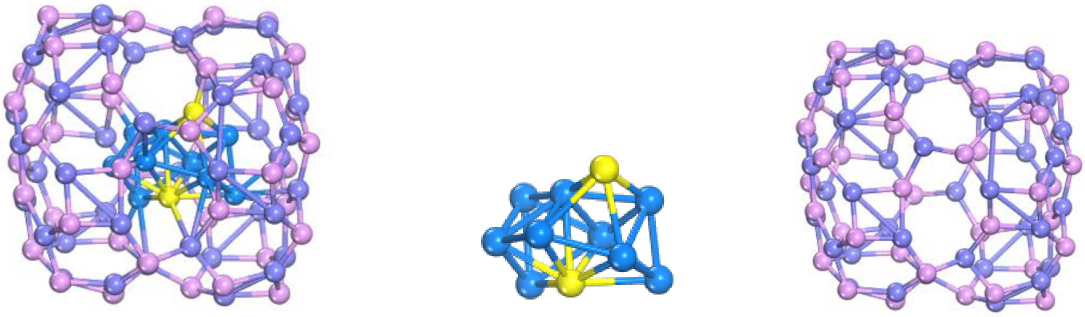

Co11

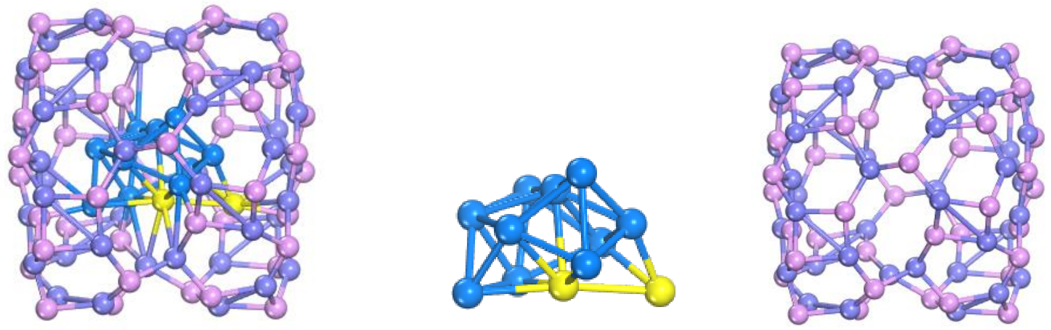

Co12

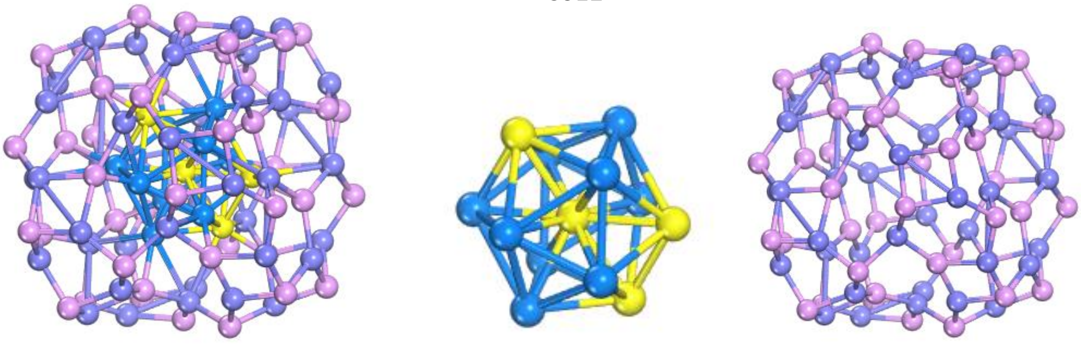

Co13

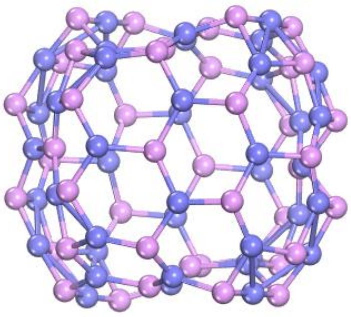

Co14

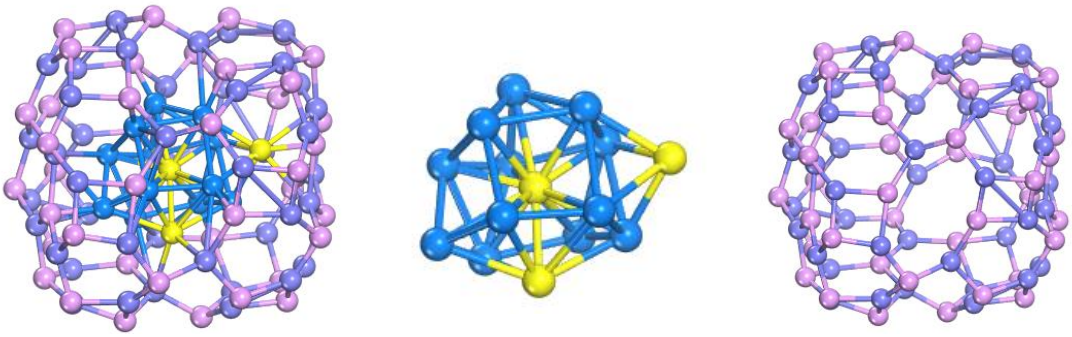

Co15

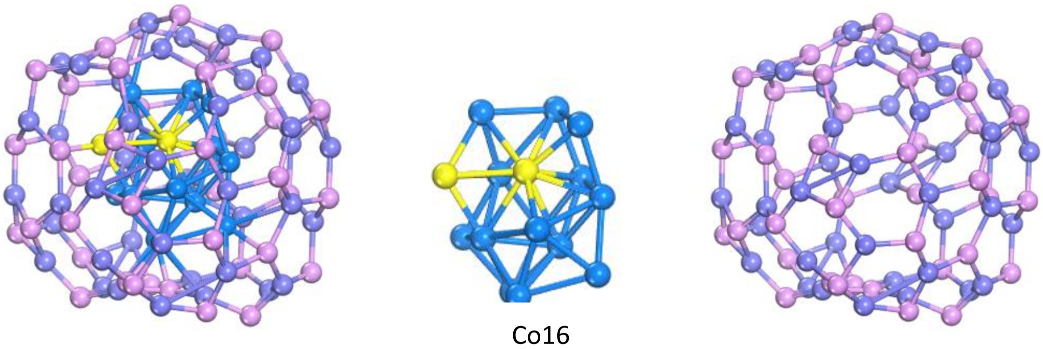

Co16

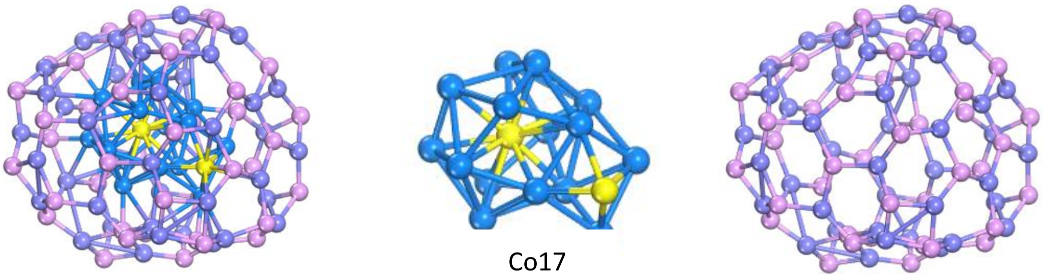

Co17

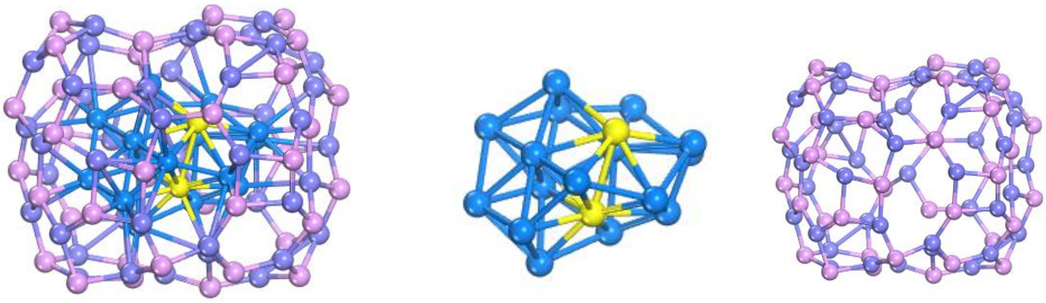

Co18

3. Ni $_n$@(ZnO)$_{42}$

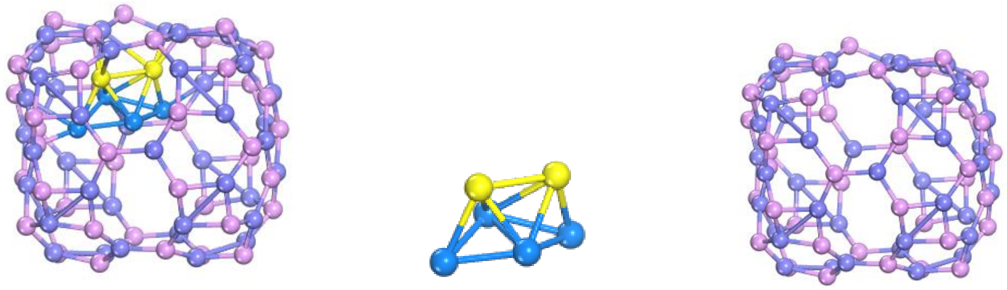

Ni6

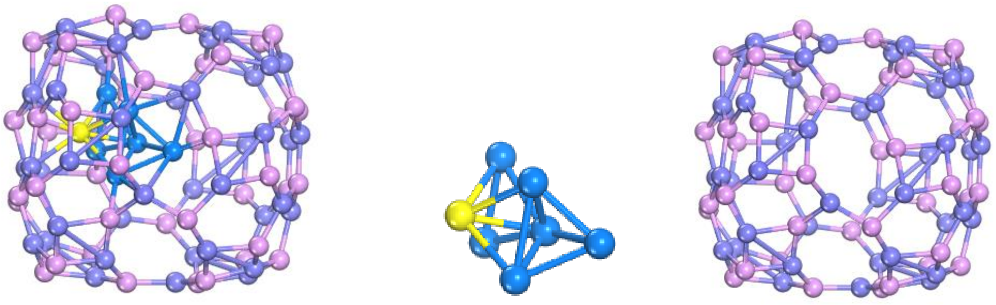

Ni7

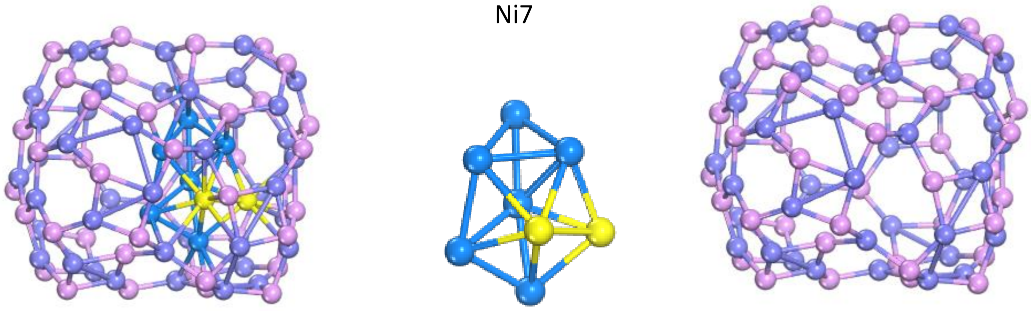

Ni8

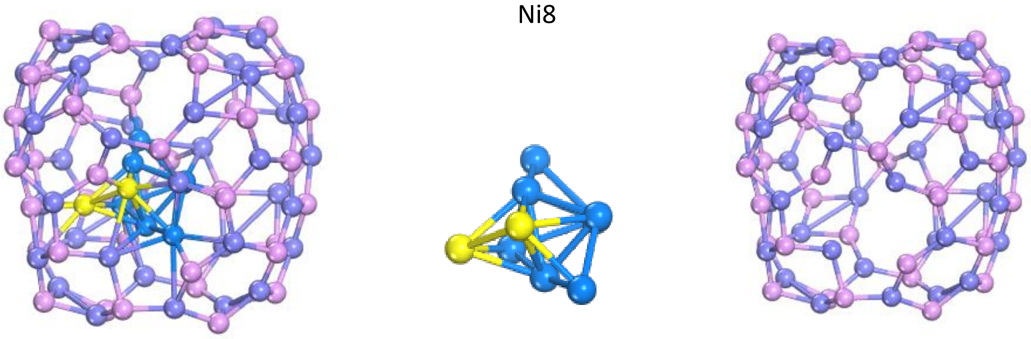

Ni9

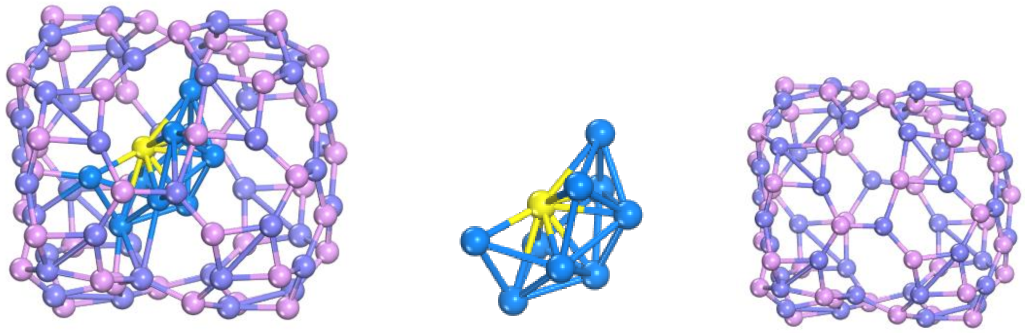

Ni10

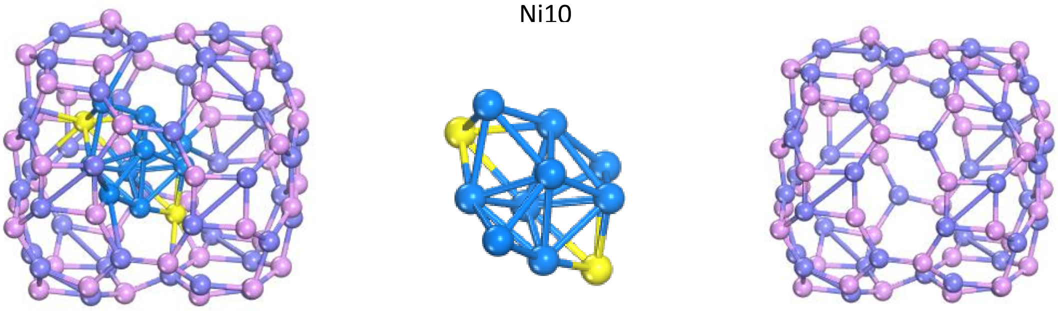

Ni11

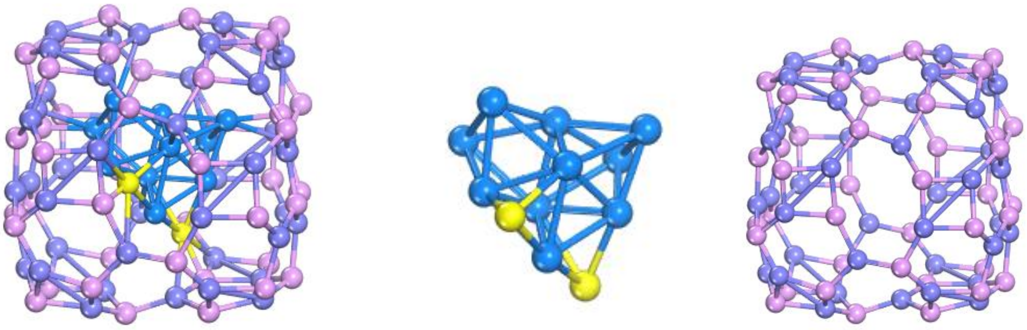

Ni12

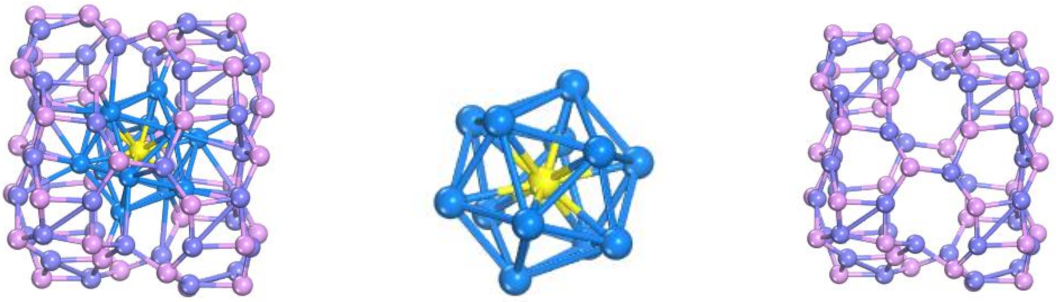

Ni13

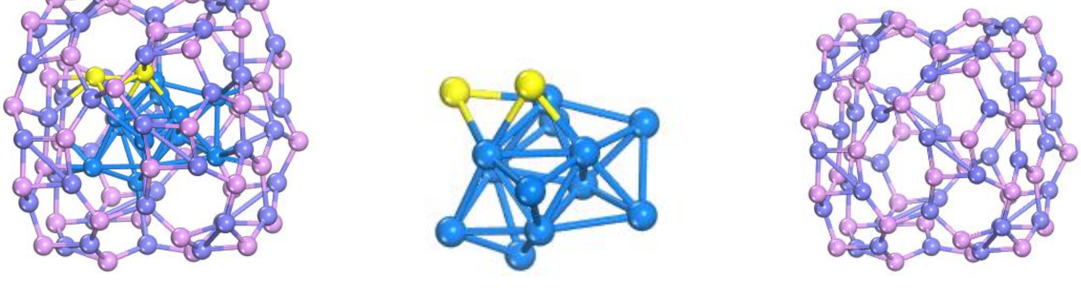

Ni14

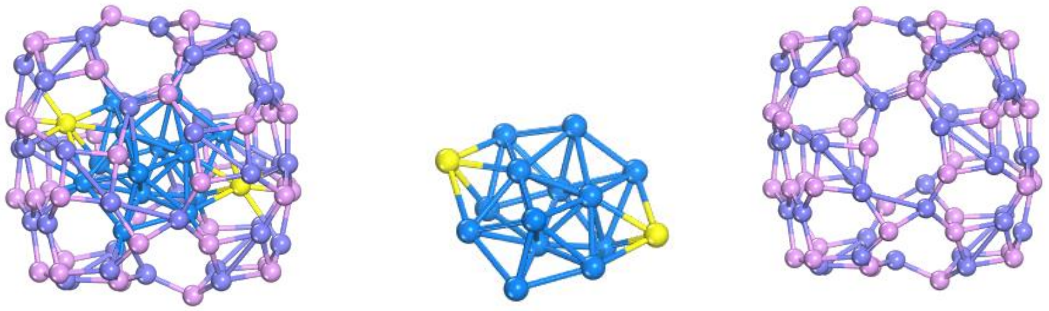

Ni15

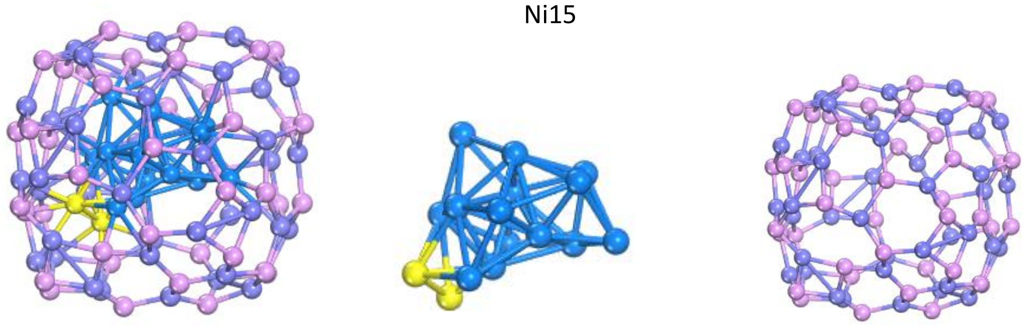

Ni16

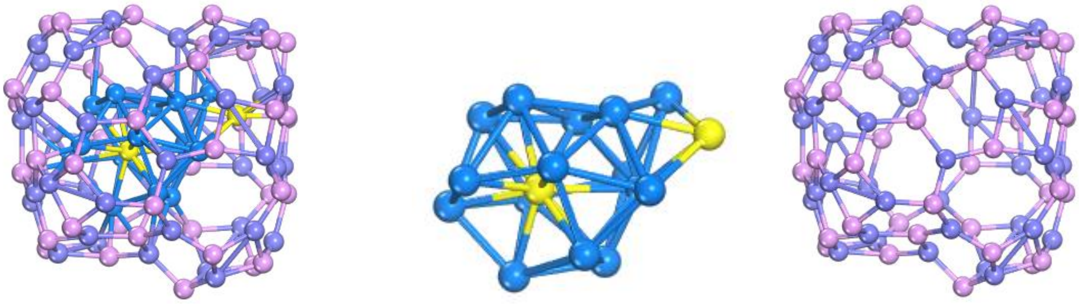

Ni17

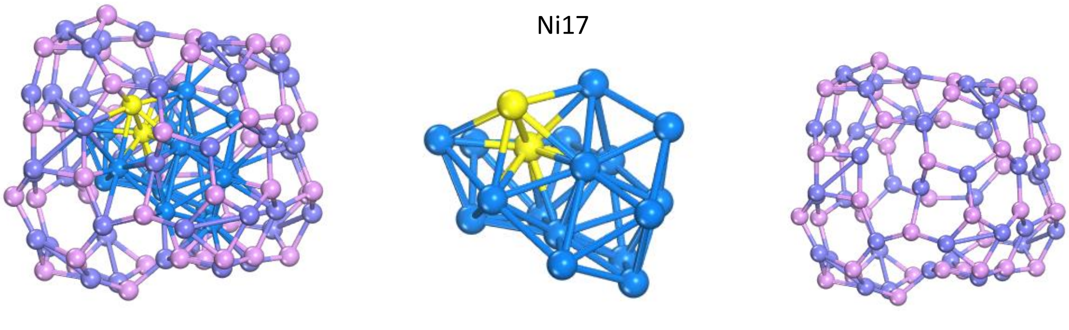

Ni18

4. Annealed $Fe_n@(ZnO)_{42}$

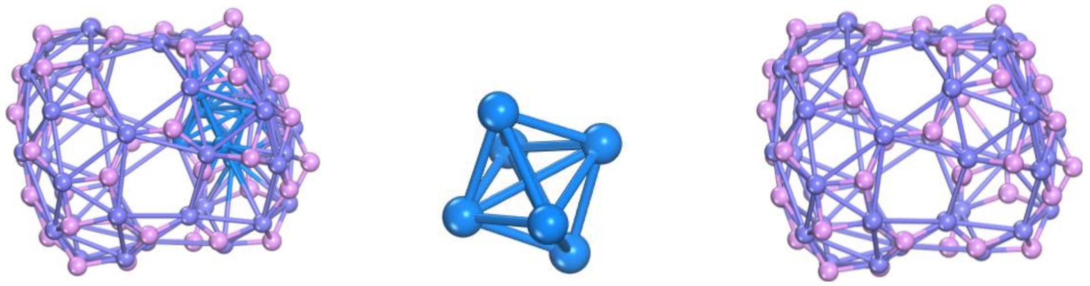

Fe6

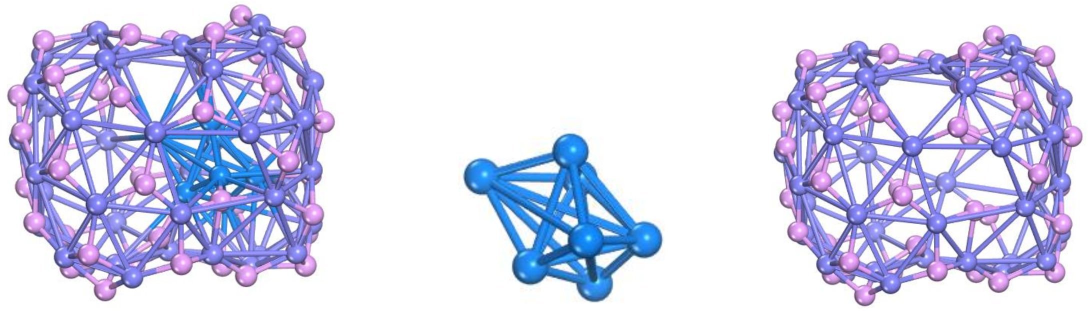

Fe7

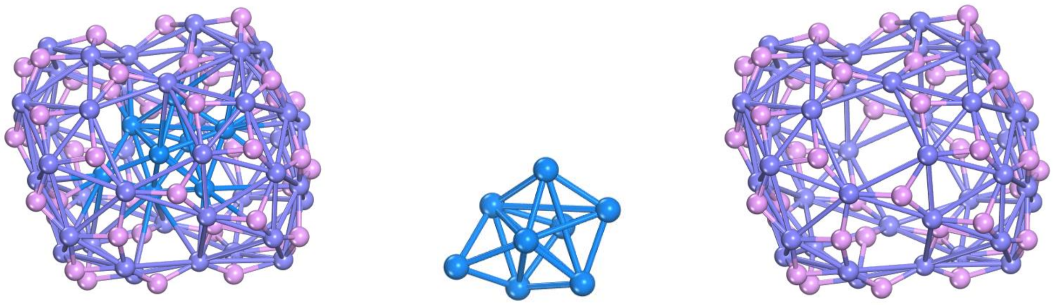

Fe8

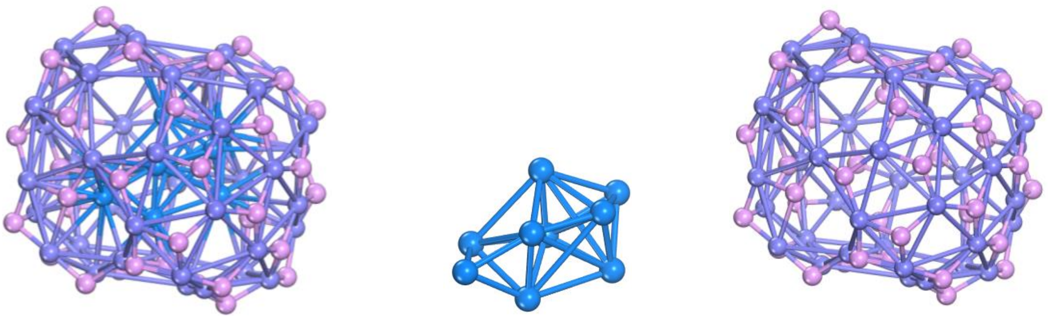

Fe9

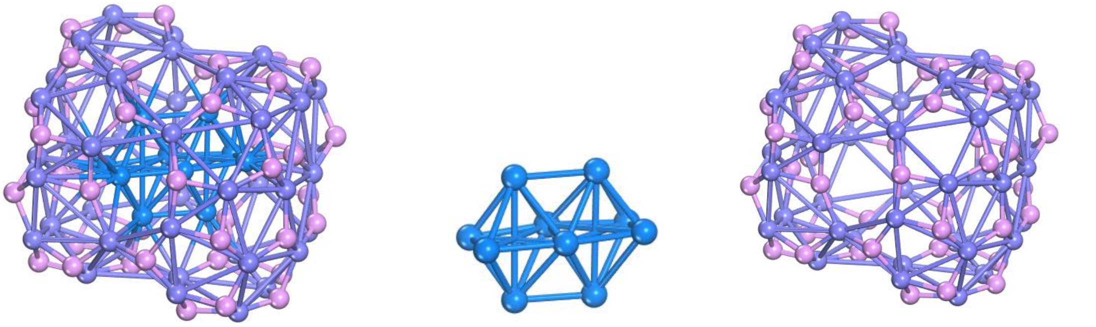

Fe10

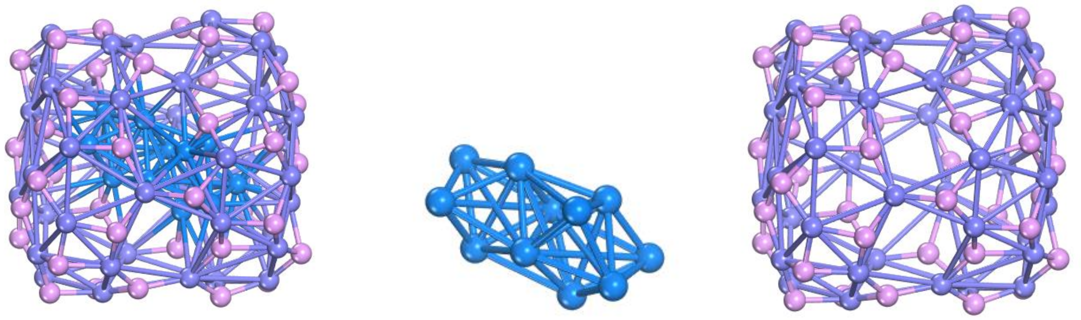

Fe11

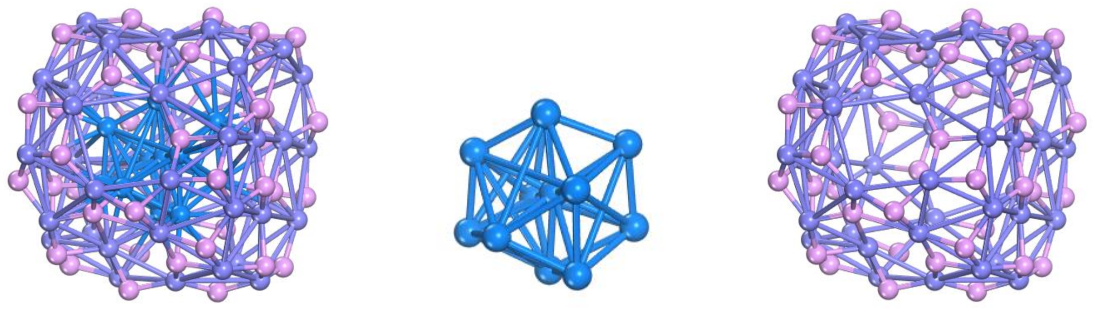

Fe12

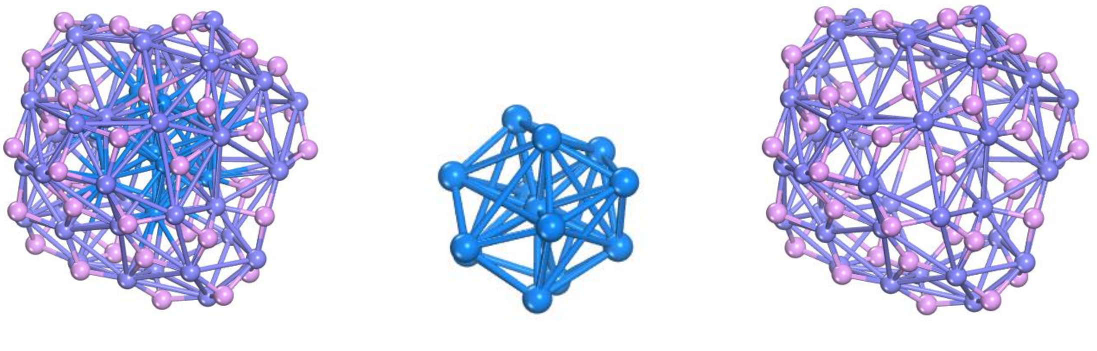

Fe13

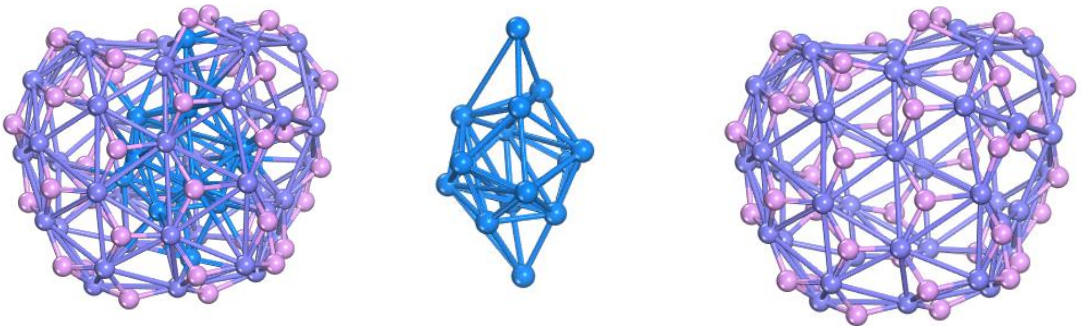

Fe14

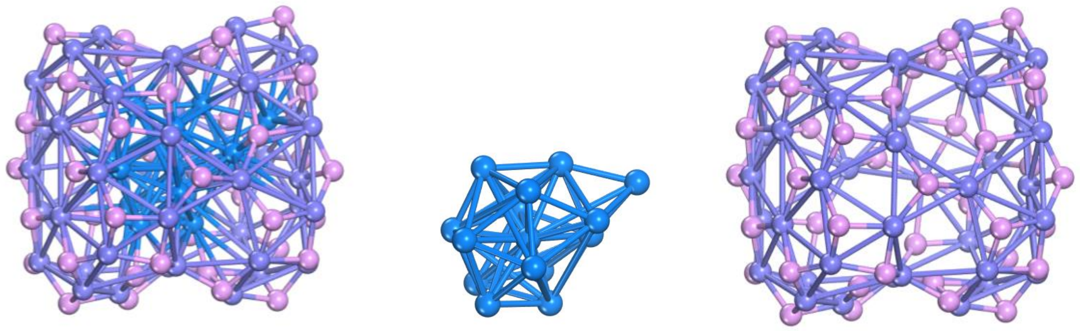

Fe15

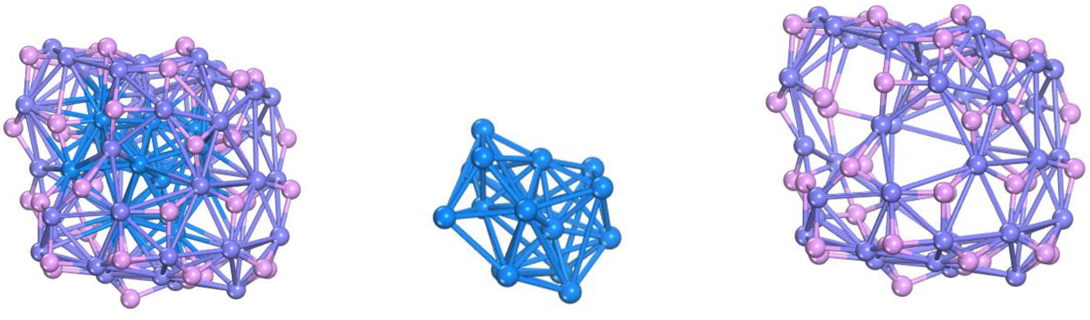

Fe16

Supporting Information II: Electric Distribution and Coordination Number

The structural, magnetic and optical properties of $TM_n@(ZnO)_{42}$ (TM = Fe, Co and Ni) hetero-nanostructure

Yaowen Hu[a], Chuting Ji[a], Xiaoxu Wang[b, c, †], Jinrong Huo[b, †], Qing Liu[b], and Yipu Song[d, *]

[a]Department of Physics, Tsinghua University, Beijing 100084, China

[b]Department of Physics, University of Science and Technology Beijing, Beijing 100083, China.

[c]Department of Cloud Platform, Beijing Computing Center, Beijing 100094, China

[d]Center for Quantum Information, IIIS, Tsinghua University, Beijing 100084, China

This Supporting information II includes the detailed result of magnetic moment, charge transfer, coordination number and some related bond length. The result are displayed in table form.

1. Related information for magnetic analysis

	$Fe_{15}@(ZnO)_{42}$									
**Number**	Fe Charge Transfer (LSDA)	Fe Charge Transfer (LSDA+U)	Fe Magnetic Moment/μB (LSDA)	Fe Magnetic Moment/μB (LSDA+U)	O Charge Transfer (LSDA)	O Charge Transfer (LSDA+U)	O Magnetic Moment/μB (LSDA)	O Magnetic Moment/μB (LSDA+U)	Zn Charge Transfer (LSDA)	Zn Charge Transfer (LSDA+U)
1	-0.2779	-0.3179	2.676	3.143	0.0072	0.0086	0.004	0.001	0.0194	0.0241
2	0.0926	0.0810	2.603	2.884	0.0018	0.0030	-0.001	-0.006	0.0125	0.0198
3	-0.0445	-0.0501	2.710	2.976	0.0052	0.0081	0.034	0.008	0.0139	0.0171
4	-0.2108	-0.2826	2.619	3.020	0.0044	0.0070	0.045	0.012	0.0146	0.0169
5	0.1431	0.1855	1.996	2.718	0.0193	0.0182	-0.006	-0.012	0.2007	0.2106
6	-0.2029	-0.2449	2.616	3.079	0.0277	0.0268	0.013	-0.019	0.1766	0.1703
7	-0.2554	-0.3067	2.772	3.194	0.0089	0.0090	-0.007	-0.019	-0.0021	0.0049
8	-0.2273	-0.3291	2.332	3.073	0.0063	0.0082	0.001	-0.009	-0.0018	-5.00E-04
9	-0.3193	-0.3530	-2.176	3.093	-0.0166	-0.0015	0.035	0.025	0.1858	0.1959
10	-0.2649	-0.2994	2.778	3.174	0.0273	0.0342	-0.009	-0.020	0.1000	0.1087
11	0.0435	0.0611	2.784	3.058	-0.0378	-0.0284	0.077	0.044	-0.0029	9.00E-04
12	-0.3207	-0.3745	2.862	3.221	-0.0130	0.0027	0.057	0.041	0.0495	0.0583
13	0.0680	0.0541	2.340	2.996	6.00E-04	9.00E-04	1.50E-02	7.00E-03	-0.0049	4.00E-04
14	-0.2247	-0.3461	-0.767	3.093	0.0110	0.0117	-0.003	-0.005	0.0819	0.0824
15	-0.2009	-0.3048	2.577	3.123	0.0038	0.0080	0.017	-0.012	0.0249	0.0264
16	-2.2021	-0.188493333			0.0074	0.0083	0.000	-0.001	0.0148	0.0195
17					-0.0292	-0.0126	0.070	0.038	0.0040	0.0091
18					0.0148	0.0142	-0.004	-0.025	0.1998	0.2350
19					0.0065	0.0090	-0.004	-0.002	0.1218	0.1403
20					0.0051	0.0061	0.002	0.000	0.0208	0.0227
21					-0.0067	-0.0057	0.042	0.012	-0.0051	-0.0035
22					-0.0125	0.0029	0.052	0.024	0.1595	0.1732
23					0.0107	0.0105	0.002	-0.005	0.0131	0.0157
24					-0.0087	-0.0053	0.042	0.021	0.0190	0.0253
25					0.0193	0.0204	0.018	0.003	0.0741	0.0820
26					-0.0179	-6.00E-04	0.043	0.037	-0.0046	7.00E-04
27					0.0014	0.0028	0.001	0.003	0.0031	0.0077
28					0.0087	0.0091	0.006	-0.003	0.0556	0.0762
29					-0.0288	-0.0026	0.082	0.074	0.0028	0.0080
30					0.0110	0.0106	0.000	-0.003	0.0255	0.0230
31					-0.0428	0.0052	-0.064	0.075	0.2133	0.2855
32					0.0019	0.0058	0.001	0.002	0.0244	0.0249
33					0.0206	0.0614	-0.108	0.056	0.0188	0.0206
34					0.0055	0.0169	0.047	0.015	0.0113	0.0160
35					0.0034	0.0039	0.001	-0.003	-0.0160	-0.0142
36					0.0116	0.0133	0.022	0.002	0.0059	0.0090
37					0.0017	0.0018	0.003	0.000	-0.0055	-0.0060
38					0.0281	0.0278	0.011	-0.003	0.2330	0.3236
39					0.0390	0.0403	0.003	-0.001	0.0043	0.0041
40					0.0087	0.0084	0.004	0.006	0.0081	0.0081
41					0.0045	0.0044	0.003	-0.004	-5.00E-04	2.80E-03
42					0.0070	0.0058	0.000	0.001	0.0063	0.0034
					0.1264	0.009014286			2.0757	0.058307143

	$Co_{15}@(ZnO)_{42}$					$Ni_{13}@(ZnO)_{42}$				
**Number**	Co Charge Transfer	Co Magnetic Moment/μB	O Charge Transfer	O Magnetic Moment/μB	Zn Charge Transfer	Ni Charge Transfer	Ni Magnetic Moment/μB	O Charge Transfer	O Magnetic Moment/μB	Zn Charge Transfer
1	0.0045	1.861	0.0046	0.000	0.0057	-0.2049	0.750	0.0048	0.000	7.00E-04
2	-0.1379	1.704	0.0046	0.000	-9.00E-04	-0.1507	0.742	0.0049	0.001	0.0074
3	-0.2377	1.852	0.0045	-0.006	0.0039	0.2155	0.226	-0.0240	0.022	0.0073
4	-0.1892	1.745	0.0118	-0.005	0.0032	0.1263	0.664	0.0065	-0.005	-0.0085
5	-0.1871	1.790	0.0237	-0.007	0.1038	-0.1272	0.663	0.0156	-0.003	0.0659
6	-0.1772	1.792	0.0032	-0.008	0.1456	-0.1082	0.649	0.0068	-0.002	0.1302
7	-0.1319	1.700	0.0014	-0.001	0.0426	-0.1328	0.664	0.0044	-0.002	0.1118
8	0.2181	1.167	-0.0346	0.041	0.0059	-0.1046	0.651	0.0063	0.001	0.0051
9	-0.1834	1.802	-0.025	0.051	0.0137	0.1254	0.663	0.0146	-0.003	0.1016
10	0.0261	1.816	0.0032	-0.002	0.0176	-0.0987	0.655	-0.0026	0.009	0.0794
11	0.1668	1.701	-1.00E-04	1.00E-03	-0.0044	-0.1399	0.741	-0.0381	0.026	-0.0126
12	-0.2232	1.761	0.0042	-0.003	-0.0022	-0.0999	0.655	1.00E-03	-7.00E-03	0.0138
13	0.0577	1.798	0.004	-0.008	0.0086	-0.1983	0.750	0.0075	-0.006	-0.0094
14	-0.1803	1.785	-0.0405	0.059	0.0713	-0.8980		0.0059	-0.006	0.0139
15	-0.1895	1.800	0.018	-0.008	-0.0033			-0.0018	0.002	-0.0044
16	-1.3642		0.0013	-0.002	0.0144			-0.0284	0.012	3.00E-04

17			-0.0377	0.059	0.0228			0.0045	0.002	0.0771
18			0.0064	-0.008	0.0282			-0.0383	0.027	0.0403
19			-0.0299	0.036	0.118			-0.0018	0.009	-0.0024
20			0.0125	-0.020	0.1544			0.0061	-0.001	0.0056
21			0.0216	-0.011	-0.0085			0.0060	0.000	0.1186
22			0.0104	-0.002	0.1222			0.0069	0.002	-0.0217
23			0.0153	-0.012	0.0079			0.0158	-0.003	1.00E-04
24			-0.0284	0.037	0.0069			-0.0292	0.034	0.0656
25			0.0098	-0.015	0.1232			-0.0299	0.034	0.0019
26			0.0142	-0.015	1.00E-04			0.0060	0.002	-0.0222
27			0.0033	0.002	9.00E-04			0.0065	0.000	0.0063
28			-7.00E-04	6.00E-03	0.0115			0.0050	-0.001	-0.0019
29			0.0269	-0.002	0.0501			-0.0414	0.027	0.0420
30			-0.022	0.029	0.0041			0.0053	0.002	1.00E-03
31			0.0051	-0.002	0.0063			-0.0284	0.012	-0.0051
32			0.0089	-0.007	6.00E-04			-4.00E-04	2.00E-03	0.0147
33			0.0037	-0.002	-0.0029			-0.0410	0.026	-0.0118
34			0.0048	-0.007	0.0353			0.0128	-0.003	0.0142
35			-0.0248	0.039	0.0234			0.0063	0.001	-0.0117
36			-0.0464	0.054	-1.00E-03			9.00E-04	-7.00E-03	0.1019
37			0.0055	-0.004	0.2045			0.0052	-0.001	0.0054
38			0.0117	-0.008	0.0037			0.0073	-0.002	0.1131
39			-0.0231	0.046	0.0261			-0.0285	0.022	-0.0046
40			0.0096	-0.008	0.0249			0.0066	-0.005	0.0069
41			0.0037	-0.004	0.0057			0.0035	0.001	0.0073
42			0.0091	-0.001	0.0162			0.0055	0.000	2.00E-04
			-0.0462		1.4101			-0.1453		1.04E+00

2. Related information for optical properties

For charge transfer, negative value represents lossing electrons and positive value represents getting electrons

	$Co_{15}@(ZnO)_{42}$					$Fe_{13}@(ZnO)_{42}$					$Ni_{15}@(ZnO)_{42}$				
**Number**	Co Charge Transfer	Co Magnetic Moment/μB	O Charge Transfer	O Magnetic Moment/μB	Zn Charge Transfer	Fe Charge Transfer	Fe Magnetic Moment/μB	O Charge Transfer	O Magnetic Moment/μB	Zn Charge Transfer	Ni Charge Transfer	Ni Magnetic Moment/μB	O Charge Transfer	O Magnetic Moment/μB	Zn Charge Transfer
1	0.0045	1.861	0.0046	0.000	0.0057	-0.1998	2.758	0.0341	-0.006	0.003	0.0642	0.563	0.0024	0.000	0.0029
2	-0.1379	1.704	0.0046	0.000	-9.00E-04	-0.1645	2.619	0.0059	0.002	0.3526	-0.0893	0.507	0.0021	0.000	0.0026
3	-0.2377	1.852	0.0045	-0.006	0.0039	-0.3036	2.748	-0.0022	0.041	5.00E-04	-0.1483	0.713	9.00E-04	-0.003	-0.0038
4	-0.1892	1.745	0.0118	-0.005	0.0032	-0.2066	2.815	0.0148	0.056	0.0037	0.1607	0.439	5.00E-04	-0.004	-0.0082
5	-0.1871	1.790	0.0237	-0.007	0.1038	-0.2185	2.563	0.0485	0.004	0.0434	-0.1025	0.511	0.0169	-0.001	0.1580
6	-0.1772	1.792	0.0032	-0.008	0.1456	-0.2782	2.813	0.0077	0.002	0.0390	-0.1351	0.511	6.00E-04	-0.001	0.0495
7	-0.1319	1.700	0.0014	-0.001	0.0426	0.2096	1.537	0.0082	-0.001	0.0168	-0.0427	0.381	-0.0420	0.024	0.0034
8	0.2181	1.167	-0.0346	0.041	0.0059	-0.2061	2.665	0.0105	-0.001	0.0611	-0.1372	0.557	0.0013	-0.002	0.0171
9	-0.1834	1.802	-0.0250	0.051	0.0137	-0.2718	2.786	0.0125	-0.001	0.0734	0.0491	0.580	0.0022	-0.002	0.0054
10	0.0261	1.816	0.0032	-0.002	0.0176	-0.2146	2.459	0.0157	0.027	0.0116	-0.0962	0.574	0.0025	-0.001	-0.0044
11	0.1668	1.701	-1.00E-04	1.00E-03	-0.0044	-0.2106	2.770	-0.0249	0.058	0.1044	-0.0944	0.517	0.0083	-0.001	0.0326
12	-0.2232	1.761	0.0042	-0.003	-0.0022	-0.2507	2.816	0.0124	-0.002	0.0038	0.0908	0.618	-0.0385	0.032	0.0089
13	0.0577	1.798	0.0040	-0.008	0.0086	-0.1983	2.544	0.0045	0.001	0.0064	-0.0567	0.423	-0.0366	0.003	0.0719
14	-0.1803	1.785	-0.0405	0.059	0.0713			-0.0065	0.034	0.1252	-0.0993	0.594	0.0028	-0.008	0.0062
15	-0.1895	1.800	0.0180	-0.008	-0.0033			0.0114	0.001	-0.0022	0.0691	0.623	0.0011	-0.002	0.0101
16			0.0013	-0.002	0.0144			0.0119	0.002	0.0086			-0.0633	0.001	0.0230
17			-0.0377	0.059	0.0228			0.0366	-0.005	0.0094			0.0050	-0.003	-0.0047
18			0.0064	-0.008	0.0282			0.0244	0.032	0.3647			-0.0435	0.010	0.0720
19			-0.0299	0.036	0.1189			-0.0211	0.064	0.0647			0.0076	-0.009	0.0718
20			0.0125	-0.020	0.1544			0.0079	-0.009	0.0151			0.0074	-0.005	-0.0094
21			0.0216	-0.011	-0.0085			-5.00E-04	0.000	0.0113			0.0047	-0.002	0.0923
22			0.0104	-0.002	0.1222			-9.00E-04	0.005	0.1381			0.0113	-0.007	0.0091
23			0.0153	-0.012	0.0079			0.0070	-0.003	-0.0058			-0.0534	0.020	-0.0127
24			-0.0284	0.037	0.0069			0.0120	0.007	0.0027			0.0149	-0.001	0.0343
25			0.0098	-0.015	0.1232			0.0053	-0.006	0.0254			0.0119	-0.006	0.0068
26			0.0142	-0.015	1.00E-04			0.0033	0.039	0.0210			0.0230	0.000	-0.0243
27			0.0033	0.002	9.00E-04			-0.0082	0.046	0.0142			0.0061	-0.001	0.0072
28			-7.00E-04	6.00E-03	0.0115			0.0057	0.003	0.0561			0.0104	-0.003	0.0558
29			0.0269	-0.002	0.0501			0.0102	-0.002	0.0161			-0.0693	0.016	-0.0137
30			-0.0220	0.029	0.0041			0.0095	-0.003	0.0101			0.0073	0.000	0.0238
31			0.0051	-0.002	0.0063			0.0024	0.071	0.3247			-0.0644	0.008	0.0285
32			0.0089	-0.007	6.00E-04			0.0046	0.003	-0.0148			0.0051	-0.002	0.0039
33			0.0037	-0.002	-0.0029			-0.0078	0.040	0.0046			-0.0683	0.027	0.0377
34			0.0048	-0.007	0.0353			0.0245	-0.006	0.0055			0.0093	-0.004	0.0067
35			-0.0248	0.039	0.0234			0.0064	0.001	0.0197			0.0021	-0.003	0.0013
36			-0.0464	0.054	-1.00E-03			0.0246	-0.017	0.1270			-0.0364	0.006	0.0389
37			0.0055	-0.004	0.2045			0.0042	0.007	0.0072			0.0055	-0.004	0.0020
38			0.0117	-0.008	0.0037			0.0070	-0.002	0.0782			0.0093	-0.006	0.0589
39			-0.0231	0.046	0.0261			-0.0174	0.039	0.0038			0.0075	-0.008	0.0017
40			0.0096	-0.008	0.0249			0.0072	0.001	0.0201			0.0118	-0.003	0.0064
41			0.0037	-0.004	0.0057			0.0120	0.004	0.0056			0.0048	0.000	-0.0013
42			0.0091	-0.001	0.0162			0.0018	0.000	0.0121			0.0050	-0.001	0.0041
Total	-1.3642	26.074	-0.0462	0.282	1.4101	-2.5137	33.893	0.3252	0.526	2.1881	-0.5678	8.111	-0.3041	0.054	0.8723

It can be seen from this table that :

1) **For Co_{15}@ZnO_{42}: Co loss electrons and the charges transfer to Zn. At the same time, O loss only a very small part of electrons, indicating that, with the introducing of Co atoms, the interaction between Co and Zn increase and the interaction between Zn and O decrease**

2) **For Fe_{13}@ZnO_{42}: Fe loss electrons. Most of the charges transfer to Zn and small part of electrons transfer to O. The interaction between Zn and O decrease.**

3) **For Ni_{15}@ZnO_{42}: A lot of electrons transfer to Zn from O. Compared with the case of Co and Fe, here the mount of electrons transfer to Zn from Ni is the smallest. So the interaction between Zn and O is much stronger in this case.**

3. Coordination Number

Co_{15}@$(ZnO)_{42}$				Fe_{15}@$(ZnO)_{42}$				Ni_{13}@$(ZnO)_{42}$			
Atom1	Atom2	bond-length	coordination number	Atom1	Atom2	bond-length	coordination number	Atom1	Atom2	bond-length	coordination number
Co1	Co8	2.2585	7	Fe1	Fe4	2.3893	6	Ni1	Ni3	2.4549	6
	Co6	2.3671			Fe2	2.4290			Ni4	2.4682	
	Co7	2.4143			Fe3	2.4544			Ni5	2.4783	
	Co10	2.4768			Fe7	2.5451			Ni10	2.5122	
	Co2	2.5097			Fe5	2.5480			Ni8	2.5398	
	Co3	2.5345			Fe6	2.8609			Ni2	2.7532	
	Co4	2.6438		average-length		2.53778333		average-length		2.534433333	
average-length		2.457814286		Fe2	Fe5	2.2642	6	Ni2	Ni3	2.4485	6
Co2	Co14	2.3695	6		Fe1	2.4290			Ni4	2.4702	
	Co13	2.4350			Fe9	2.4992			Ni8	2.5134	
	Co8	2.4615			Fe7	2.5771			Ni12	2.5136	
	Co6	2.4667			Fe8	2.5963			Ni7	2.5441	
	Co4	2.4943			Fe3	2.6519			Ni1	2.7532	
	Co1	2.5097		average-length		2.50295		average-length		2.5405	
average-length		2.456116667		Fe3	Fe8	2.2564	6	Ni3	Ni4	2.3001	12
Co3	Co10	2.2490	5		Fe6	2.3295			Ni9	2.3017	
	Co4	2.3536			Fe1	2.4544			Ni10	2.3932	
	Co5	2.4177			Fe5	2.5277			Ni12	2.3936	
	Co1	2.5345			Fe2	2.6519			Ni8	2.4120	
	Co8	2.6136			Fe10	2.8701			Ni6	2.4147	
average-length		2.43368		average-length		2.515			Ni5	2.4428	
Co4	Co3	2.3536	6	Fe4	Fe1	2.3893	6		Ni7	2.4433	
	Co13	2.4071			Fe6	2.4317			Ni11	2.4470	
	Co5	2.4822			Fe15	2.4430			Ni2	2.4485	
	Co2	2.4943			Fe5	2.4746			Ni1	2.4549	
	Co8	2.5008			Fe11	2.5642			Ni13	2.4552	
	Co1	2.6438			Fe7	2.7598		average-length		2.408916667	
average-length		2.4803		average-length		2.51043333		Ni4	Ni3	2.3001	6
Co5	Co15	2.3685	6	Fe5	Fe2	2.2642	13		Ni12	2.4261	
	Co11	2.3773			Fe12	2.3673			Ni1	2.4682	
	Co3	2.4177			Fe11	2.4644			Ni5	2.4702	
	Co8	2.4761			Fe4	2.4746			Ni2	2.4702	
	Co4	2.4822			Fe10	2.5049			Ni6	2.6296	
	Co13	2.5596			Fe13	2.5254		average-length		2.460733333	
average-length		2.4469			Fe3	2.5277		Ni5	Ni3	2.4428	6
Co6	Co1	2.3671	6		Fe1	2.5480			Ni4	2.4702	
	Co7	2.3709			Fe6	2.5705			Ni1	2.4783	
	Co2	2.4667			Fe7	2.6108			Ni11	2.5458	
	Co14	2.4695			Fe15	2.6292			Ni6	2.5638	
	Co8	2.4833			Fe8	2.6618			Ni10	2.6754	
	Co9	2.6215			Fe9	2.6777		average-length		2.529383333	
average-length		2.463166667		average-length		2.52511538		Ni6	Ni3	2.4147	6
Co7	Co10	2.2748	4	Fe6	Fe3	2.3295	6		Ni12	2.4867	
	Co6	2.3709			Fe11	2.4189			Ni11	2.5132	
	Co1	2.4143			Fe4	2.4317			Ni13	2.5397	
	Co9	2.4590			Fe5	2.5705			Ni5	2.5638	
average-length		2.37975			Fe10	2.6066			Ni4	2.6296	
Co8	Co1	2.2585	13		Fe1	2.8609		average-length		2.524616667	
	Co11	2.2801		average-length		2.53635		Ni7	Ni3	2.4433	6
	Co13	2.3067		Fe7	Fe13	2.4375	7		Ni9	2.4723	
	Co14	2.4255			Fe14	2.4918			Ni13	2.4803	
	Co10	2.4262			Fe1	2.5451			Ni2	2.5441	
	Co2	2.4615			Fe2	2.5771			Ni8	2.5622	
	Co5	2.4761			Fe5	2.6108			Ni12	2.6654	
	Co12	2.4790			Fe4	2.7598		average-length		2.527933333	
	Co6	2.4833			Fe15	2.8131		Ni8	Ni3	2.4120	6
	Co4	2.5008		average-length		2.60502857			Ni10	2.4852	
	Co9	2.5463		Fe8	Fe3	2.2564	5		Ni2	2.5134	
	Co3	2.6136			Fe9	2.3254			Ni1	2.5398	
	Co15	2.7733			Fe10	2.5062			Ni7	2.5622	
average-length		2.463915385			Fe2	2.5963			Ni9	2.6247	
Co9	Co10	2.2815	6		Fe5	2.6618		average-length		2.522883333	
	Co12	2.3078		average-length		2.46922		Ni9	Ni3	2.3017	6
	Co7	2.4590		Fe9	Fe13	2.2915	5		Ni10	2.4225	
	Co8	2.5463			Fe8	2.3254			Ni13	2.4616	

	Co11	2.6037			Fe2	2.4992			Ni7	2.4723	
	Co6	2.6215			Fe5	2.6777			Ni11	2.4741	
average-length		2.469966667			Fe12	2.7466			Ni8	2.6247	
Co10	Co3	2.2490	6	average-length		2.50808		average-length		2.459483333	
	Co7	2.2748		Fe10	Fe11	2.4889	6	Ni10	Ni3	2.3932	6
	Co9	2.2815			Fe12	2.4997			Ni9	2.4225	
	Co8	2.4262			Fe5	2.5049			Ni8	2.4852	
	Co11	2.4325			Fe8	2.5062			Ni1	2.5122	
	Co1	2.4768			Fe6	2.6066			Ni11	2.5144	
average-length		2.3568			Fe3	2.8701			Ni5	2.6754	
Co11	Co8	2.2801	6	average-length		2.5794		average-length		2.500483333	
	Co5	2.3773		Fe11	Fe6	2.4189	6	Ni11	Ni3	2.4470	6
	Co12	2.4226			Fe15	2.4400			Ni9	2.4741	
	Co10	2.4325			Fe12	2.4642			Ni6	2.5132	
	Co15	2.5693			Fe5	2.4644			Ni10	2.5144	
	Co9	2.6037			Fe10	2.4889			Ni5	2.5458	
average-length		2.447583333			Fe4	2.5642			Ni13	2.7429	
Co12	Co15	2.3011	5	average-length		2.47343333		average-length		2.539566667	
	Co9	2.3078		Fe12	Fe5	2.3673	6	Ni12	Ni3	2.3936	6
	Co14	2.3826			Fe11	2.4642			Ni4	2.4261	
	Co11	2.4226			Fe10	2.4997			Ni6	2.4867	
	Co8	2.4790			Fe13	2.6182			Ni2	2.5136	
average-length		2.37862			Fe9	2.7466			Ni13	2.5181	
Co13	Co15	2.2507	6		Fe15	2.7717			Ni7	2.6654	
	Co8	2.3067		average-length		2.57795		average-length		2.500583333	
	Co4	2.4071		Fe13	Fe14	2.2202	6	Ni13	Ni3	2.4552	6
	Co14	2.4176			Fe9	2.2915			Ni9	2.4616	
	Co2	2.4350			Fe7	2.4375			Ni7	2.4803	
	Co5	2.5596			Fe5	2.5254			Ni12	2.5181	
average-length		2.396116667			Fe15	2.5408			Ni6	2.5397	
Co14	Co2	2.3695	5		Fe12	2.6182			Ni11	2.7429	
	Co12	2.3826		average-length		2.43893333		average-length		2.532966667	
	Co13	2.4176		Fe14	Fe13	2.2202	3				
	Co8	2.4255			Fe15	2.4614					
	Co6	2.4695			Fe7	2.4918					
average-length		2.41294		average-length		2.39113333					
Co15	Co13	2.2507	5	Fe15	Fe11	2.4400	7				
	Co12	2.3011			Fe4	2.4430					
	Co5	2.3685			Fe14	2.4614					
	Co11	2.5693			Fe13	2.5408					
	Co8	2.7733			Fe5	2.6292					
average-length		2.45258			Fe12	2.7717					
					Fe7	2.8131					
				average-length		2.5856					

Supporting Information III: Bond length of TM@ZnO

The structural, magnetic and optical properties of $TM_n@(ZnO)_{42}$ (TM = Fe, Co and Ni) hetero-nanostructure

Yaowen Hu[a], Chuting Ji[a], Xiaoxu Wang[b, c, †], Jinrong Huo[b, †], Qing Liu[b], and Yipu Song[d, *]

[a]Department of Physics, Tsinghua University, Beijing 100084, China

[b]Department of Physics, University of Science and Technology Beijing, Beijing 100083, China.

[c]Department of Cloud Platform, Beijing Computing Center, Beijing 100094, China

[d]Center for Quantum Information, IIIS, Tsinghua University, Beijing 100084, China

This Supporting information I includes the detailed result of bond length. The results are displayed in table form.

1. Bond Length information of $Fe_n@ZnO_{42}$

$Fe_6@(ZnO)_{42}$			
Number	Atom1	Atom2	Length
1	Fe1	Fe2	2.2967
2	Fe1	Fe3	2.5269
3	Fe1	Fe4	2.4935
4	Fe1	Fe5	2.3500
5	Fe2	Fe3	2.3043
6	Fe2	Fe4	2.2869
7	Fe2	Fe5	2.9573
8	Fe2	Fe6	2.3563
9	Fe3	Fe5	2.5533
10	Fe3	Fe6	2.4971
11	Fe4	Fe5	2.393
12	Fe4	Fe6	2.8084
13	Fe5	Fe6	2.2696
average bond length			2.468715

$Fe_7@(ZnO)_{42}$			
Number	Atom1	Atom2	Length
1	Fe1	Fe2	2.3917
2	Fe1	Fe4	2.4380
3	Fe1	Fe6	2.3537
4	Fe2	Fe3	2.3168
5	Fe2	Fe4	2.4029
6	Fe2	Fe6	2.8223
7	Fe2	Fe7	2.6856
8	Fe3	Fe4	2.9526
9	Fe3	Fe5	2.3302
10	Fe3	Fe6	2.3159
11	Fe3	Fe7	2.3137
12	Fe4	Fe5	2.2956
13	Fe4	Fe6	2.5191
14	Fe4	Fe7	2.4715
15	Fe5	Fe6	2.5148
16	Fe5	Fe7	2.4684
average bond length			2.47455

$Fe_8@(ZnO)_{42}$			
Number	Atom1	Atom2	Length
1	Fe1	Fe2	2.3692
2	Fe1	Fe3	2.5182
3	Fe1	Fe6	2.7262
4	Fe1	Fe7	2.5213
5	Fe1	Fe8	2.3165
6	Fe2	Fe3	2.5294
7	Fe2	Fe4	2.3091
8	Fe2	Fe5	2.6928
9	Fe2	Fe6	2.3465
10	Fe2	Fe7	2.6617
11	Fe3	Fe4	2.3999
12	Fe3	Fe7	2.319
13	Fe4	Fe5	2.3229
14	Fe4	Fe7	2.8033
15	Fe5	Fe6	2.4723
16	Fe5	Fe7	2.3165
17	Fe6	Fe7	2.5131
18	Fe6	Fe8	2.4454
19	Fe7	Fe8	2.4581
average bond length			2.475863

$Fe_9@(ZnO)_{42}$			
Number	Atom1	Atom2	Length
1	Fe1	Fe2	2.3940
2	Fe1	Fe3	2.5375
3	Fe1	Fe4	2.4100
4	Fe1	Fe5	2.5565
5	Fe1	Fe6	2.4211
6	Fe1	Fe7	2.6579
7	Fe1	Fe8	2.5141
8	Fe2	Fe3	2.4259
9	Fe2	Fe7	2.3134
10	Fe2	Fe8	2.3086
11	Fe2	Fe9	2.4205
12	Fe3	Fe4	2.4288
13	Fe3	Fe5	2.5191
14	Fe3	Fe7	2.6751
15	Fe4	Fe5	2.2919
16	Fe5	Fe6	2.5590
17	Fe5	Fe7	2.3186
18	Fe6	Fe7	2.3484
19	Fe6	Fe8	2.4343
20	Fe7	Fe8	2.5747
21	Fe7	Fe9	2.3833
22	Fe8	Fe9	2.5519
average bond length			2.456573

$Fe_{10}@(ZnO)_{42}$			
Number	Atom1	Atom2	Length
1	Fe1	Fe2	2.7955
2	Fe1	Fe3	2.7059
3	Fe1	Fe4	2.4200
4	Fe1	Fe5	2.8382
5	Fe1	Fe6	2.6503
6	Fe1	Fe8	2.3056
7	Fe1	Fe9	2.3992
8	Fe2	Fe3	2.5298
9	Fe2	Fe8	2.4393
10	Fe2	Fe9	2.4067
11	Fe2	Fe10	2.4389
12	Fe3	Fe4	2.4473
13	Fe3	Fe7	2.5377
14	Fe3	Fe8	2.4040
15	Fe4	Fe5	2.3611
16	Fe4	Fe7	2.3292
17	Fe4	Fe8	2.9283
18	Fe5	Fe6	2.4226
19	Fe5	Fe7	2.5961
20	Fe5	Fe8	2.4229
21	Fe6	Fe8	2.4089
22	Fe6	Fe9	2.4324
23	Fe6	Fe10	2.8813
24	Fe7	Fe8	2.4349
25	Fe8	Fe9	2.8857
26	Fe8	Fe10	2.4314
27	Fe9	Fe10	2.3018
average bond length			2.524259

$Fe_{11}@(ZnO)_{42}$			
Number	Atom1	Atom2	Length
1	Fe1	Fe2	2.4574
2	Fe1	Fe3	2.4386
3	Fe1	Fe4	2.7767
4	Fe1	Fe5	2.6443
5	Fe1	Fe6	2.4154
6	Fe1	Fe8	2.3046
7	Fe1	Fe9	2.5142
8	Fe1	Fe11	2.7030
9	Fe2	Fe3	2.8829
10	Fe2	Fe4	2.3663
11	Fe2	Fe9	2.4165
12	Fe2	Fe10	2.3011
13	Fe2	Fe11	2.9305
14	Fe3	Fe4	2.3520
15	Fe3	Fe5	2.4284
16	Fe3	Fe7	2.3874
17	Fe3	Fe8	2.9482
18	Fe4	Fe7	2.7165
19	Fe4	Fe8	2.4647
20	Fe4	Fe10	2.9440
21	Fe4	Fe11	2.3223
22	Fe5	Fe7	2.4892
23	Fe5	Fe8	2.3298
24	Fe6	Fe8	2.7075
25	Fe6	Fe9	2.5473
26	Fe6	Fe11	2.3136
27	Fe7	Fe8	2.4009
28	Fe8	Fe11	2.4839
29	Fe9	Fe10	2.5843
30	Fe9	Fe11	2.5091
31	Fe10	Fe11	2.3129
average bond length			2.528823

$Fe_{12}@(ZnO)_{42}$			
Number	Atom1	Atom2	Length
1	Fe1	Fe2	2.5667
2	Fe1	Fe3	2.4503
3	Fe1	Fe4	2.4621
4	Fe1	Fe6	2.4408
5	Fe2	Fe3	2.2657
6	Fe2	Fe4	2.4238
7	Fe2	Fe5	2.9354
8	Fe2	Fe10	2.4679
9	Fe2	Fe11	2.4090
10	Fe3	Fe4	2.5278
11	Fe3	Fe5	2.3561
12	Fe3	Fe6	2.4147
13	Fe3	Fe7	2.4734
14	Fe3	Fe8	2.6956
15	Fe3	Fe9	2.2820
16	Fe3	Fe10	2.4233
17	Fe3	Fe11	2.7054
18	Fe3	Fe12	2.5035
19	Fe4	Fe5	2.5402
20	Fe4	Fe8	2.5333
21	Fe5	Fe8	2.5835
22	Fe5	Fe11	2.4895
23	Fe5	Fe12	2.7828
24	Fe6	Fe8	2.4667
25	Fe6	Fe9	2.5314
26	Fe7	Fe9	2.4303
27	Fe7	Fe10	2.5410
28	Fe7	Fe12	2.4911
29	Fe8	Fe9	2.4419
30	Fe9	Fe12	2.4306
31	Fe10	Fe11	2.4306
32	Fe10	Fe12	3.0398
33	Fe11	Fe12	2.3564
average bond length			2.511897

$Fe_{13}@(ZnO)_{42}$			
Number	Atom1	Atom2	Length
1	Fe1	Fe3	2.4834
2	Fe1	Fe4	2.8531
3	Fe1	Fe5	2.4225
4	Fe1	Fe7	2.4348
5	Fe1	Fe8	2.4484
6	Fe1	Fe9	2.9846
7	Fe2	Fe3	2.4932
8	Fe2	Fe4	2.4091
9	Fe2	Fe7	2.6831
10	Fe2	Fe11	2.4354
11	Fe2	Fe12	2.3993
12	Fe3	Fe7	2.3610
13	Fe3	Fe8	2.5923
14	Fe3	Fe12	2.4898
15	Fe4	Fe5	2.4023
16	Fe4	Fe7	2.3845
17	Fe4	Fe11	2.5494
18	Fe5	Fe6	2.4426
19	Fe5	Fe7	2.6197
20	Fe5	Fe9	2.4795
21	Fe6	Fe7	2.3838
22	Fe6	Fe9	2.5413
23	Fe6	Fe11	2.6065
24	Fe6	Fe13	2.5671
25	Fe7	Fe8	2.5315
26	Fe7	Fe9	2.4794
27	Fe7	Fe10	2.4239
28	Fe7	Fe11	2.5137
29	Fe7	Fe12	2.4081
30	Fe7	Fe13	2.5817
31	Fe8	Fe9	2.4310
32	Fe8	Fe10	2.4175
33	Fe9	Fe10	2.4961
34	Fe10	Fe12	2.8468
35	Fe10	Fe13	2.2856
36	Fe11	Fe12	2.8991
37	Fe11	Fe13	2.3464
38	Fe12	Fe13	2.4992
average bond length			2.516492

$Fe_{14}@(ZnO)_{42}$			
Number	Atom1	Atom2	Length
1	Fe1	Fe2	2.4239
2	Fe1	Fe4	2.3310
3	Fe1	Fe7	2.4915
4	Fe1	Fe8	2.3665
5	Fe1	Fe10	2.5690
6	Fe2	Fe3	2.6019
7	Fe2	Fe4	2.4696
8	Fe2	Fe6	2.4364
9	Fe2	Fe7	2.5560
10	Fe2	Fe13	2.4065
11	Fe3	Fe4	2.5081
12	Fe3	Fe6	2.8856
13	Fe4	Fe5	2.6307
14	Fe4	Fe6	2.3502
15	Fe4	Fe10	2.3614
16	Fe5	Fe6	2.5745
17	Fe5	Fe10	2.3909
18	Fe5	Fe12	2.4086
19	Fe5	Fe14	2.6157
20	Fe6	Fe13	2.4851
21	Fe6	Fe14	2.3385
22	Fe7	Fe8	2.5333
23	Fe7	Fe9	2.4146
24	Fe7	Fe13	2.4623
25	Fe8	Fe9	2.5838
26	Fe8	Fe10	2.4648
27	Fe8	Fe11	2.5868
28	Fe8	Fe12	2.3262
29	Fe9	Fe11	2.6280
30	Fe9	Fe12	2.3946
31	Fe9	Fe13	2.5196
32	Fe9	Fe14	2.3074
33	Fe10	Fe12	2.5248
34	Fe11	Fe12	2.4306
35	Fe12	Fe14	2.5117
36	Fe13	Fe14	2.4981
average bond length			2.483006

$Fe_{15}@(ZnO)_{42}$			
Number	Atom1	Atom2	Length
1	Fe1	Fe2	2.4290
2	Fe1	Fe3	2.4544
3	Fe1	Fe4	2.3893
4	Fe1	Fe5	2.5480
5	Fe1	Fe6	2.8609
6	Fe1	Fe7	2.5451
7	Fe2	Fe3	2.6519
8	Fe2	Fe5	2.2642
9	Fe2	Fe7	2.5771
10	Fe2	Fe8	2.5963
11	Fe2	Fe9	2.4992
12	Fe2	Fe13	3.0486
13	Fe3	Fe5	2.5277
14	Fe3	Fe6	2.3295
15	Fe3	Fe8	2.2564
16	Fe3	Fe10	2.8701
17	Fe4	Fe5	2.4746
18	Fe4	Fe6	2.4317
19	Fe4	Fe7	2.7598
20	Fe4	Fe11	2.5642
21	Fe4	Fe15	2.4430
22	Fe5	Fe6	2.5705
23	Fe5	Fe7	2.6108
24	Fe5	Fe8	2.6618
25	Fe5	Fe9	2.6777
26	Fe5	Fe10	2.5049
27	Fe5	Fe11	2.4644
28	Fe5	Fe12	2.3673
29	Fe5	Fe13	2.5254
30	Fe5	Fe15	2.6292
31	Fe6	Fe10	2.6066
32	Fe6	Fe11	2.4189
33	Fe7	Fe13	2.4375
34	Fe7	Fe14	2.4917
35	Fe7	Fe15	2.8131
36	Fe8	Fe9	2.3254
37	Fe8	Fe10	2.5062
38	Fe9	Fe12	2.7466
39	Fe9	Fe13	2.2915
40	Fe10	Fe11	2.4889
41	Fe10	Fe12	2.4997
42	Fe11	Fe12	2.4642
43	Fe11	Fe15	2.4400
44	Fe12	Fe13	2.6182
45	Fe12	Fe15	2.7717
46	Fe13	Fe14	2.2202
47	Fe13	Fe15	2.5408
48	Fe14	Fe15	2.4614
average bond length			2.534908

$Fe_{16}@(ZnO)_{42}$			
Number	Atom1	Atom2	Length
1	Fe1	Fe2	2.3747
2	Fe1	Fe3	2.4732
3	Fe1	Fe4	2.4318
4	Fe1	Fe5	2.8509
5	Fe1	Fe9	2.4392
6	Fe2	Fe3	2.9923
7	Fe2	Fe5	2.3621
8	Fe2	Fe6	2.3955
9	Fe2	Fe8	2.5264
10	Fe2	Fe9	2.7575
11	Fe2	Fe13	2.4832
12	Fe2	Fe15	2.6572
13	Fe3	Fe4	2.6788
14	Fe3	Fe5	2.3414
15	Fe3	Fe13	2.366
16	Fe3	Fe14	2.6497
17	Fe4	Fe5	2.3664
18	Fe4	Fe9	2.4459
19	Fe4	Fe11	3.0864
20	Fe5	Fe8	2.4686
21	Fe5	Fe9	2.3999
22	Fe5	Fe11	2.3321
23	Fe5	Fe13	2.7708
24	Fe5	Fe14	2.2687
25	Fe5	Fe15	2.6208
26	Fe5	Fe16	2.5228
27	Fe6	Fe7	2.4144
28	Fe6	Fe8	2.3953
29	Fe7	Fe8	2.6142
30	Fe7	Fe9	2.3726
31	Fe7	Fe12	2.3591
32	Fe8	Fe9	2.5147
33	Fe8	Fe10	2.3723
34	Fe8	Fe11	2.6278
35	Fe8	Fe12	2.5528
36	Fe8	Fe15	2.4529
37	Fe8	Fe16	2.8200
38	Fe9	Fe11	2.5966
39	Fe9	Fe12	2.4694
40	Fe10	Fe11	2.6186
41	Fe10	Fe12	2.4748
42	Fe10	Fe16	2.8125
43	Fe11	Fe12	2.3787
44	Fe11	Fe16	2.4607
45	Fe13	Fe14	2.3872
46	Fe13	Fe15	2.2940
47	Fe14	Fe15	2.7320
48	Fe14	Fe16	2.4936
49	Fe15	Fe16	2.4086
average bond length			2.524186

2. Bond Length information of $Co_n@ZnO_{42}$

$Co_6@(ZnO)_{42}$

Atom1	Atom2	Length
Co1	Co2	2.3259
Co1	Co3	2.2487
Co1	Co4	2.3431
Co1	Co5	2.3700
Co2	Co3	2.2566
Co2	Co4	2.3372
Co2	Co5	2.3925
Co2	Co6	2.2387
Co3	Co5	2.4409
Co4	Co5	2.3538
Co4	Co6	2.3549
Co5	Co6	2.3509
average bond length		2.334433

$Co_7@(ZnO)_{42}$

Atom1	Atom2	Length
Co1	Co2	2.3644
Co1	Co3	2.2848
Co1	Co4	2.6181
Co1	Co6	2.3798
Co1	Co7	2.2541
Co2	Co3	2.3484
Co2	Co5	2.3233
Co2	Co6	2.3301
Co3	Co4	2.3564
Co3	Co5	2.4908
Co3	Co7	2.3508
Co4	Co7	2.2449
Co5	Co6	2.3475
Co5	Co7	2.3115
Co6	Co7	2.3423
average bond length		2.35648

$Co_8@(ZnO)_{42}$

Atom1	Atom2	Length
Co1	Co2	2.4038
Co1	Co3	2.3411
Co1	Co4	2.3900
Co1	Co6	2.5118
Co1	Co7	2.3122
Co1	Co8	2.2927
Co2	Co3	2.3477
Co2	Co5	2.3493
Co2	Co6	2.3868
Co3	Co4	2.2648
Co3	Co5	2.2843
Co3	Co7	2.3765
Co4	Co7	2.3818
Co5	Co6	2.4628
Co5	Co7	2.3783
Co6	Co7	2.3452
Co6	Co8	2.4259
Co7	Co8	2.3810
average bond length		2.368667

$Co_9@(ZnO)_{42}$

Atom1	Atom2	Length
Co1	Co2	2.3313
Co1	Co3	2.3050
Co1	Co4	2.4067
Co1	Co7	2.3823
Co1	Co8	2.3075
Co1	Co9	2.2984
Co2	Co3	2.9350
Co2	Co5	2.3084
Co2	Co6	2.4526
Co2	Co7	2.3867
Co2	Co8	2.5779
Co3	Co4	2.4161
Co3	Co5	2.3712
Co3	Co7	2.3834
Co4	Co7	2.3476
Co4	Co9	2.4374
Co5	Co7	2.3903
Co6	Co7	2.3198
Co6	Co8	2.2939
Co6	Co9	2.5489
Co7	Co9	2.3930
Co8	Co9	2.3572
average bond length		2.406845

$Co_{10}@(ZnO)_{42}$

Atom1	Atom2	Length
Co1	Co2	2.3796
Co1	Co3	2.3761
Co1	Co4	2.4461
Co1	Co5	2.3841
Co1	Co7	2.2571
Co1	Co8	2.4352
Co1	Co10	2.4032
Co2	Co3	2.5507
Co2	Co7	2.3873
Co2	Co8	2.3580
Co2	Co9	2.2754
Co3	Co4	2.4105
Co3	Co6	2.3373
Co3	Co7	2.4006
Co4	Co5	2.2767
Co4	Co6	2.3294
Co5	Co6	2.3534
Co5	Co7	2.3796
Co5	Co10	2.5493
Co6	Co7	2.4319
Co7	Co9	2.4434
Co7	Co10	2.3743
Co8	Co9	2.3273
Co8	Co10	2.3341
Co9	Co10	2.4140
average bond length		2.384584

$Co_{11}@(ZnO)_{42}$

Atom1	Atom2	Length
Co1	Co2	2.4560
Co1	Co4	2.5240
Co1	Co6	2.3651
Co1	Co7	2.5077
Co1	Co9	2.3619
Co2	Co3	2.3721
Co2	Co4	2.3380
Co2	Co5	2.3773
Co2	Co6	2.4302
Co2	Co9	2.3270
Co2	Co10	2.4490
Co2	Co11	3.1075
Co3	Co4	2.3024
Co3	Co5	2.3756
Co3	Co8	2.3602
Co4	Co6	2.2878
Co4	Co8	2.3333
Co5	Co6	2.3178
Co5	Co8	2.4001
Co5	Co10	2.3462
Co5	Co11	2.5533
Co6	Co7	2.3704
Co6	Co8	2.3816
Co6	Co9	2.9517
Co6	Co11	2.3299
Co7	Co9	2.4514
Co7	Co11	2.2938
Co9	Co10	2.4847
Co9	Co11	2.3044
Co10	Co11	2.2637
average bond length		2.424137

$Co_{12}@(ZnO)_{42}$

Atom1	Atom2	Length
Co1	Co2	2.4668
Co1	Co3	2.4141
Co1	Co4	2.4057
Co1	Co5	2.3512
Co1	Co6	2.3541
Co1	Co7	2.5104
Co1	Co9	3.1042
Co1	Co11	2.5595
Co1	Co12	2.4332
Co2	Co3	2.3115
Co2	Co5	2.5322
Co2	Co9	2.2823
Co3	Co4	2.3490
Co3	Co7	2.3169
Co3	Co8	2.4146
Co3	Co9	2.5363
Co4	Co8	2.3813
Co4	Co12	2.3215
Co5	Co6	2.3644
Co5	Co7	3.0519
Co5	Co9	2.3049
Co5	Co10	2.4203
Co6	Co7	2.3336
Co6	Co10	2.4290
Co6	Co11	2.3199
Co6	Co12	2.5649
Co7	Co8	2.4178
Co7	Co9	2.3148
Co7	Co10	2.3972
Co7	Co12	2.3304
Co8	Co12	2.3004
Co9	Co10	2.3025
Co11	Co12	2.2110
average bond length		2.427509

$Co_{13}@(ZnO)_{42}$

Atom1	Atom2	Length
Co1	Co2	2.6227
Co1	Co3	2.3645
Co1	Co4	2.4706
Co1	Co5	2.4455
Co1	Co7	2.4423
Co1	Co9	2.4653
Co2	Co3	2.3854
Co2	Co4	2.4872
Co2	Co8	2.4769
Co2	Co9	2.4153
Co2	Co12	2.5522
Co3	Co4	2.4003
Co3	Co5	2.3751
Co3	Co6	2.4099
Co3	Co7	2.3647
Co3	Co8	2.3741
Co3	Co9	2.4087
Co3	Co10	2.4017
Co3	Co11	2.3848
Co3	Co12	2.3640
Co3	Co13	2.3638
Co4	Co5	2.4845
Co4	Co6	2.7126
Co4	Co12	2.4358
Co5	Co6	2.4820
Co5	Co7	2.6992
Co5	Co11	2.4780
Co6	Co11	2.4140
Co6	Co12	2.4313
Co6	Co13	2.4649
Co7	Co9	2.4291
Co7	Co10	2.4335
Co7	Co11	2.5580
Co8	Co9	2.4827
Co8	Co10	2.4867
Co8	Co12	2.6948
Co8	Co13	2.4459
Co9	Co10	2.7131
Co10	Co11	2.4879
Co10	Co13	2.4728
Co11	Co13	2.6161
Co12	Co13	2.4435
average bond length		2.472414

$Co_{14}@(ZnO)_{42}$

Atom1	Atom2	Length
Co1	Co2	2.4394
Co1	Co4	2.3276
Co1	Co5	2.4933
Co1	Co8	2.4077
Co1	Co9	2.2930
Co1	Co12	2.4351
Co2	Co3	2.5639
Co2	Co5	2.3807
Co2	Co7	2.3667
Co2	Co8	2.3821
Co2	Co11	2.6156
Co2	Co12	2.3983
Co2	Co13	2.4658
Co2	Co14	2.3168
Co3	Co5	2.4216
Co3	Co7	2.2670
Co4	Co5	2.2593
Co4	Co6	2.9179
Co4	Co12	2.3334
Co5	Co6	2.3171
Co5	Co7	2.5924
Co5	Co12	2.7040
Co6	Co7	2.3698
Co6	Co12	2.3508
Co6	Co13	2.4084
Co7	Co13	2.2751
Co8	Co9	2.3423
Co8	Co11	2.3392
Co9	Co10	2.2833
Co9	Co11	2.5867
Co9	Co12	2.3477
Co10	Co11	2.4314
Co10	Co12	2.5447
Co11	Co12	2.3978
Co11	Co13	2.4863
Co11	Co14	2.2614
Co12	Co13	2.3826
Co13	Co14	2.3688
average bond length		2.417763

$Co_{15}@(ZnO)_{42}$

Atom1	Atom2	Length
Co1	Co2	2.5097
Co1	Co3	2.5345
Co1	Co4	2.6438
Co1	Co6	2.3671
Co1	Co7	2.4143
Co1	Co8	2.2585
Co1	Co10	2.4768
Co2	Co4	2.4943
Co2	Co6	2.4667
Co2	Co8	2.4615
Co2	Co13	2.4350
Co2	Co14	2.3695
Co3	Co4	2.3536
Co3	Co5	2.4177
Co3	Co8	2.6136
Co3	Co10	2.2490
Co3	Co11	3.0252
Co4	Co5	2.4822
Co4	Co8	2.5008
Co4	Co13	2.4071
Co5	Co8	2.4761
Co5	Co11	2.3773
Co5	Co13	2.5596
Co5	Co15	2.3685
Co6	Co7	2.3709
Co6	Co8	2.4833
Co6	Co9	2.6215
Co6	Co14	2.4695
Co7	Co9	2.4590
Co7	Co10	2.2748
Co8	Co9	2.5463
Co8	Co10	2.4262
Co8	Co11	2.2801
Co8	Co12	2.4790
Co8	Co13	2.3067
Co8	Co14	2.4255
Co8	Co15	2.7733
Co9	Co10	2.2815
Co9	Co11	2.6037
Co9	Co12	2.3078
Co10	Co11	2.4325
Co11	Co12	2.4226
Co11	Co15	2.5693
Co12	Co14	2.3826
Co12	Co15	2.3011
Co13	Co14	2.4176
Co13	Co15	2.2507
average bond length		2.449955

$Co_{16}@(ZnO)_{42}$

Atom1	Atom2	Length
Co1	Co2	2.3384
Co1	Co3	2.3903
Co1	Co4	2.3881
Co1	Co9	2.3155
Co2	Co3	2.3270
Co2	Co5	2.3579
Co2	Co6	2.2515
Co2	Co8	2.4852
Co2	Co9	2.5446
Co2	Co13	2.3711
Co2	Co15	2.6522
Co3	Co4	2.3777
Co3	Co5	2.2945
Co3	Co13	2.5972
Co4	Co5	2.4267
Co4	Co9	2.3698
Co4	Co11	2.5474
Co5	Co8	2.6493
Co5	Co11	2.2977
Co5	Co13	2.4701
Co5	Co14	2.2941
Co5	Co15	2.5054
Co5	Co16	2.4361
Co6	Co7	2.3290
Co6	Co8	2.4555
Co6	Co9	2.5760
Co7	Co8	2.4216
Co7	Co9	2.3075
Co7	Co12	2.3523
Co8	Co9	2.3298
Co8	Co10	2.2619
Co8	Co11	2.4069
Co8	Co12	2.6067
Co8	Co15	2.3018
Co8	Co16	2.5026
Co9	Co11	2.5546
Co9	Co12	2.6838
Co10	Co11	2.8271
Co10	Co12	2.3529
Co10	Co16	2.2963
Co11	Co12	2.2244
Co11	Co16	2.3795
Co13	Co14	2.5135
Co13	Co15	2.2786
Co14	Co15	2.3608
Co14	Co16	2.3773
Co15	Co16	2.3731
average bond length		2.420453

$Co_{17}@(ZnO)_{42}$

Atom1	Atom2	Length
Co1	Co2	2.3204
Co1	Co3	2.4064
Co1	Co4	2.4301
Co1	Co5	2.4052
Co1	Co8	2.8009
Co1	Co9	2.5768
Co2	Co3	2.3356
Co2	Co4	2.4022
Co2	Co6	2.5307
Co2	Co11	2.4902
Co3	Co4	2.4656
Co3	Co5	2.7881
Co3	Co6	2.5201
Co3	Co7	2.4110
Co3	Co8	2.3856
Co3	Co9	2.4178
Co3	Co10	2.4951
Co3	Co11	2.5219
Co3	Co12	2.3735
Co3	Co13	2.5461
Co3	Co14	2.7493
Co4	Co5	2.3711
Co4	Co6	2.4365
Co4	Co7	2.3530
Co5	Co7	2.8072
Co5	Co9	2.2893
Co5	Co14	2.3189
Co6	Co7	2.3810
Co6	Co11	2.2826
Co6	Co12	2.6437
Co7	Co12	2.5065
Co7	Co14	2.3149
Co7	Co15	2.3028
Co7	Co16	2.3447
Co8	Co9	2.2910
Co8	Co10	2.6592
Co8	Co13	2.4541
Co9	Co10	2.3534
Co9	Co14	2.3446
Co10	Co12	2.6169
Co10	Co13	2.3194
Co10	Co14	2.4277
Co10	Co17	2.4343
Co11	Co12	2.5968
Co11	Co13	2.2862
Co12	Co13	2.4182
Co12	Co16	2.2893
Co12	Co17	2.5801
Co14	Co15	2.2737
Co14	Co17	2.3048
Co15	Co16	2.3031
Co15	Co17	2.5406
Co16	Co17	2.3917
average bond length		2.44547

$Co_{18}@(ZnO)_{42}$

Atom1	Atom2	Length
Co1	Co2	2.3860
Co1	Co3	2.3381
Co1	Co4	2.4102
Co1	Co6	2.4298
Co1	Co8	2.4078
Co1	Co9	2.3551
Co1	Co11	2.5741
Co1	Co13	2.4100
Co2	Co6	2.5998
Co2	Co8	2.5955
Co2	Co11	2.5162
Co2	Co16	2.3091
Co3	Co4	2.4171
Co3	Co5	2.5356
Co3	Co9	2.4146
Co3	Co13	2.3931
Co4	Co5	2.3487
Co4	Co6	2.4014
Co4	Co7	2.2494
Co5	Co6	2.6629
Co5	Co13	2.3151
Co5	Co14	2.6027
Co6	Co7	2.3219
Co6	Co11	2.4859
Co6	Co13	2.8992
Co6	Co14	2.2876
Co6	Co15	2.3481
Co6	Co16	2.4472
Co6	Co18	2.6710
Co7	Co15	2.3405
Co8	Co9	2.5219
Co8	Co10	2.3325
Co8	Co11	2.3262
Co9	Co10	2.2789
Co9	Co13	2.3470
Co10	Co11	2.3413
Co10	Co12	2.4323
Co10	Co13	2.3813
Co11	Co12	2.4445
Co11	Co13	2.3912
Co11	Co14	2.5700
Co11	Co16	2.3510
Co11	Co17	2.3229
Co12	Co13	2.4623
Co12	Co14	2.3501
Co12	Co17	2.4539
Co13	Co14	2.5010
Co14	Co17	2.3652
Co14	Co18	2.3866
Co15	Co16	2.3434
Co15	Co18	2.3406
Co16	Co17	2.3788
Co16	Co18	2.2789
Co17	Co18	2.4417
average bond length		2.422541

3. Bond Length information of $Ni_n@ZnO_{42}$

$Ni_6@(ZnO)_{42}$		
Atom1	Atom2	Length
Ni1	Ni2	2.4181
Ni1	Ni3	2.3952
Ni1	Ni6	2.3851
Ni2	Ni3	2.3042
Ni2	Ni4	2.5702
Ni2	Ni5	2.4434
Ni2	Ni6	2.5954
Ni3	Ni4	2.3397
Ni3	Ni5	2.3309
Ni3	Ni6	2.3247
Ni4	Ni5	2.3623
Ni5	Ni6	2.3912
average bond length		2.405033

$Ni_7@(ZnO)_{42}$		
Atom1	Atom2	Length
Ni1	Ni3	2.3023
Ni1	Ni4	2.4598
Ni1	Ni5	2.5470
Ni1	Ni6	2.3848
Ni2	Ni3	2.3333
Ni2	Ni4	2.4967
Ni2	Ni7	2.3808
Ni3	Ni4	2.3166
Ni3	Ni5	2.2965
Ni3	Ni6	2.5423
Ni3	Ni7	2.3892
Ni4	Ni5	2.4260
Ni4	Ni7	2.3665
Ni5	Ni6	2.3421
Ni5	Ni7	2.9683
average bond length		2.436813

$Ni_8@(ZnO)_{42}$		
Atom1	Atom2	Length
Ni1	Ni2	2.5448
Ni1	Ni3	2.5214
Ni1	Ni4	2.3350
Ni1	Ni6	2.5537
Ni1	Ni7	2.4700
Ni2	Ni3	2.3729
Ni2	Ni4	2.4178
Ni2	Ni5	2.5388
Ni2	Ni7	2.4282
Ni2	Ni8	2.4408
Ni3	Ni4	2.3867
Ni3	Ni5	2.3757
Ni4	Ni5	2.3629
Ni4	Ni6	2.3988
Ni4	Ni7	2.3713
Ni4	Ni8	2.3642
Ni5	Ni8	2.4466
Ni6	Ni7	2.4004
Ni7	Ni8	2.4784
average bond length		2.432021

$Ni_9@(ZnO)_{42}$		
Atom1	Atom2	Length
Ni1	Ni2	2.6213
Ni1	Ni4	2.4860
Ni1	Ni6	2.3620
Ni1	Ni7	2.4358
Ni1	Ni8	2.4410
Ni2	Ni3	2.3866
Ni2	Ni4	2.3351
Ni2	Ni5	2.4731
Ni2	Ni6	2.3683
Ni2	Ni8	2.3900
Ni2	Ni9	2.3844
Ni3	Ni4	2.3709
Ni3	Ni5	2.3803
Ni4	Ni5	2.3638
Ni4	Ni6	2.3592
Ni5	Ni6	2.4807
Ni5	Ni9	2.6154
Ni6	Ni7	2.3325
Ni6	Ni8	2.3642
Ni6	Ni9	2.3729
Ni7	Ni8	2.4946
Ni8	Ni9	2.4791
average bond length		2.4226

$Ni_{10}@(ZnO)_{42}$		
Atom1	Atom2	Length
Ni1	Ni2	2.4761
Ni1	Ni4	2.3138
Ni1	Ni5	2.4624
Ni1	Ni6	2.3083
Ni1	Ni8	2.4978
Ni1	Ni9	2.3645
Ni2	Ni3	2.4084
Ni2	Ni4	2.4888
Ni2	Ni8	2.3272
Ni2	Ni9	2.3857
Ni2	Ni10	2.4439
Ni3	Ni4	2.3079
Ni3	Ni7	2.5331
Ni3	Ni8	2.5748
Ni3	Ni10	3.3818
Ni4	Ni5	2.4407
Ni4	Ni7	2.3300
Ni4	Ni8	2.4173
Ni5	Ni6	2.3896
Ni5	Ni7	2.4324
Ni5	Ni8	2.2960
Ni6	Ni8	2.5447
Ni6	Ni9	2.5584
Ni7	Ni8	2.5080
Ni8	Ni9	2.7197
Ni8	Ni10	2.3307
Ni9	Ni10	2.3826
average bond length		2.467578

$Ni_{11}@(ZnO)_{42}$		
Atom1	Atom2	Length
Ni1	Ni2	2.4271
Ni1	Ni4	2.3253
Ni1	Ni5	3.1578
Ni1	Ni7	2.4258
Ni1	Ni8	2.3701
Ni1	Ni10	2.3311
Ni1	Ni11	2.3335
Ni2	Ni3	2.4722
Ni2	Ni4	2.3898
Ni2	Ni5	2.3360
Ni2	Ni8	2.3678
Ni2	Ni9	2.3692
Ni2	Ni11	2.9518
Ni3	Ni4	2.3002
Ni3	Ni5	2.3824
Ni3	Ni6	2.6870
Ni4	Ni5	2.4656
Ni4	Ni6	2.3197
Ni4	Ni7	2.3471
Ni5	Ni6	2.3932
Ni5	Ni7	2.3917
Ni5	Ni9	2.4960
Ni5	Ni11	2.4198
Ni6	Ni7	2.4646
Ni7	Ni10	2.3773
Ni7	Ni11	2.4051
Ni8	Ni9	2.4662
Ni8	Ni11	2.4767
Ni9	Ni11	2.3275
Ni10	Ni11	2.3427
average bond length		2.44401

$Ni_{12}@(ZnO)_{42}$		
Atom1	Atom2	Length
Ni1	Ni2	2.4738
Ni1	Ni3	2.3496
Ni1	Ni4	2.3506
Ni1	Ni5	2.3461
Ni1	Ni8	2.8439
Ni1	Ni10	2.3512
Ni1	Ni11	2.4774
Ni1	Ni12	2.8380
Ni2	Ni3	2.5227
Ni2	Ni4	2.5222
Ni2	Ni8	2.2938
Ni3	Ni5	3.0489
Ni3	Ni6	2.4814
Ni3	Ni7	2.3688
Ni3	Ni8	2.3890
Ni3	Ni10	2.5672
Ni3	Ni12	2.3686
Ni4	Ni5	2.5482
Ni4	Ni8	2.3321
Ni4	Ni9	3.4341
Ni5	Ni6	2.4822
Ni5	Ni8	2.3741
Ni5	Ni9	2.3650
Ni5	Ni11	2.5195
Ni5	Ni12	2.3882
Ni6	Ni7	2.4150
Ni6	Ni8	2.3410
Ni6	Ni9	2.4170
Ni6	Ni12	2.3430
Ni7	Ni12	2.3198
Ni8	Ni9	2.3186
Ni10	Ni11	2.5156
Ni10	Ni12	2.3293
Ni11	Ni12	2.2957
average bond length		2.480341

$Ni_{13}@(ZnO)_{42}$		
Atom1	Atom2	Length
Ni1	Ni2	2.7532
Ni1	Ni3	2.4549
Ni1	Ni4	2.4682
Ni1	Ni5	2.4783
Ni1	Ni8	2.5398
Ni1	Ni10	2.5122
Ni2	Ni3	2.4485
Ni2	Ni4	2.4702
Ni2	Ni7	2.5441
Ni2	Ni8	2.5134
Ni2	Ni12	2.5136
Ni3	Ni4	2.3001
Ni3	Ni5	2.4428
Ni3	Ni6	2.4147
Ni3	Ni7	2.4433
Ni3	Ni8	2.4120
Ni3	Ni9	2.3017
Ni3	Ni10	2.3932
Ni3	Ni11	2.4470
Ni3	Ni12	2.3936
Ni3	Ni13	2.4552
Ni4	Ni5	2.4702
Ni4	Ni6	2.6296
Ni4	Ni12	2.4261
Ni5	Ni6	2.5638
Ni5	Ni10	2.6754
Ni5	Ni11	2.5458
Ni6	Ni11	2.5132
Ni6	Ni12	2.4867
Ni6	Ni13	2.5397
Ni7	Ni8	2.5622
Ni7	Ni9	2.4723
Ni7	Ni12	2.6654
Ni7	Ni13	2.4803
Ni8	Ni9	2.6247
Ni8	Ni10	2.4852
Ni9	Ni10	2.4225
Ni9	Ni11	2.4741
Ni9	Ni13	2.4616
Ni10	Ni11	2.5144
Ni11	Ni13	2.7429
Ni12	Ni13	2.5181
average bond length		2.499386

$Ni_{14}@(ZnO)_{42}$		
Atom1	Atom2	Length
Ni1	Ni2	2.4455
Ni1	Ni3	2.3993
Ni1	Ni5	2.4325
Ni1	Ni10	2.3722
Ni2	Ni3	2.3907
Ni2	Ni4	2.3721
Ni2	Ni6	3.2907
Ni2	Ni7	2.3809
Ni2	Ni8	2.4909
Ni2	Ni9	2.3531
Ni2	Ni10	2.4070
Ni2	Ni13	2.5298
Ni3	Ni4	2.4538
Ni3	Ni5	2.3976
Ni3	Ni6	2.3654
Ni3	Ni10	2.8519
Ni3	Ni12	2.6710
Ni3	Ni13	2.4319
Ni4	Ni9	2.8558
Ni4	Ni13	2.3133
Ni5	Ni6	2.4887
Ni5	Ni10	2.3588
Ni6	Ni8	2.4704
Ni6	Ni10	2.3547
Ni6	Ni11	2.3823
Ni6	Ni12	2.3828
Ni6	Ni13	2.5461
Ni6	Ni14	2.3597
Ni7	Ni8	2.4031
Ni7	Ni9	3.2411
Ni7	Ni10	2.3492
Ni7	Ni11	3.4075
Ni8	Ni9	2.4653
Ni8	Ni10	2.4731
Ni8	Ni11	2.3820
Ni8	Ni13	2.3633
Ni8	Ni14	2.5671
Ni9	Ni13	2.3370
Ni10	Ni11	2.3488
Ni11	Ni14	3.2975
Ni12	Ni13	2.3311
Ni12	Ni14	2.5302
Ni13	Ni14	2.3665
average bond length		2.521202

$Ni_{15}@(ZnO)_{42}$		
Atom1	Atom2	Length
Ni1	Ni2	2.5668
Ni1	Ni3	2.5205
Ni1	Ni4	2.4194
Ni1	Ni6	2.3961
Ni1	Ni7	2.4440
Ni1	Ni9	2.3844
Ni1	Ni10	2.4534
Ni1	Ni11	2.3185
Ni1	Ni12	2.4598
Ni2	Ni4	2.4343
Ni2	Ni6	2.5727
Ni2	Ni8	2.3573
Ni2	Ni9	2.4755
Ni2	Ni14	2.5231
Ni3	Ni4	2.4601
Ni3	Ni5	2.4986
Ni3	Ni11	2.4782
Ni3	Ni12	2.3636
Ni4	Ni5	2.3695
Ni4	Ni9	2.4663
Ni4	Ni12	2.5482
Ni4	Ni13	2.3804
Ni4	Ni14	2.5269
Ni4	Ni15	2.4471
Ni5	Ni12	2.4488
Ni5	Ni13	2.4189
Ni5	Ni15	2.3975
Ni6	Ni7	2.4671
Ni6	Ni8	2.5494
Ni6	Ni9	2.4099
Ni6	Ni10	2.6468
Ni7	Ni10	2.3264
Ni7	Ni11	2.5681
Ni8	Ni9	2.3138
Ni8	Ni14	2.4937
Ni9	Ni10	2.4006
Ni9	Ni12	2.4572
Ni9	Ni14	2.4152
Ni9	Ni15	2.3595
Ni10	Ni11	2.6161
Ni10	Ni12	2.3786
Ni11	Ni12	2.5448
Ni12	Ni15	2.4008
Ni13	Ni14	2.5668
Ni13	Ni15	2.3280
Ni14	Ni15	2.4719
average bond length		2.453143

$Ni_{16}@(ZnO)_{42}$		
Atom1	Atom2	Length
Ni1	Ni2	2.3786
Ni1	Ni3	2.4833
Ni1	Ni4	2.3979
Ni1	Ni5	2.5216
Ni1	Ni6	2.6200
Ni1	Ni7	2.5297
Ni1	Ni8	2.5129
Ni1	Ni9	2.4473
Ni1	Ni11	2.4690
Ni2	Ni3	2.4279
Ni2	Ni6	2.5368
Ni2	Ni7	2.4365
Ni2	Ni9	2.4258
Ni2	Ni14	2.3554
Ni2	Ni16	2.5079
Ni3	Ni4	2.4818
Ni3	Ni6	2.3283
Ni3	Ni14	2.5877
Ni4	Ni5	2.4684
Ni4	Ni6	2.5681
Ni4	Ni11	2.4341
Ni5	Ni8	2.5009
Ni5	Ni11	2.3619
Ni5	Ni12	2.4493
Ni6	Ni9	2.4835
Ni6	Ni11	2.2940
Ni6	Ni13	2.4496
Ni6	Ni14	2.3818
Ni6	Ni15	2.3139
Ni6	Ni16	2.5219
Ni7	Ni8	2.7256
Ni7	Ni9	2.3147
Ni8	Ni9	2.3944
Ni8	Ni10	2.4324
Ni8	Ni11	2.4750
Ni8	Ni12	2.5366
Ni9	Ni10	2.4276
Ni9	Ni11	2.3733
Ni9	Ni13	2.4038
Ni9	Ni16	2.3809
Ni10	Ni11	2.4056
Ni10	Ni12	2.4382
Ni10	Ni13	2.5550
Ni11	Ni12	2.3023
Ni11	Ni13	2.6023
Ni13	Ni15	2.5819
Ni13	Ni16	2.4130
Ni14	Ni15	2.4612
Ni14	Ni16	2.3750
Ni15	Ni16	2.4848
average bond length		2.455188

$Ni_{17}@(ZnO)_{42}$		
Atom1	Atom2	Length
Ni1	Ni2	2.2927
Ni1	Ni3	2.7278
Ni1	Ni4	2.5498
Ni1	Ni7	2.5487
Ni1	Ni8	2.6998
Ni1	Ni13	2.326
Ni2	Ni3	2.5381
Ni2	Ni4	2.5917
Ni2	Ni5	2.4081
Ni2	Ni6	3.3686
Ni2	Ni7	2.4552
Ni2	Ni9	2.5363
Ni2	Ni10	2.3813
Ni2	Ni11	2.3395
Ni2	Ni12	2.5085
Ni3	Ni4	2.3474
Ni3	Ni6	2.4326
Ni3	Ni7	3.4158
Ni3	Ni8	2.4014
Ni3	Ni9	2.2990
Ni3	Ni11	2.8750
Ni3	Ni13	2.3825
Ni3	Ni14	2.3540
Ni3	Ni15	2.4425
Ni3	Ni16	2.5992
Ni3	Ni17	2.4287
Ni4	Ni5	2.4776
Ni4	Ni6	2.5618
Ni4	Ni13	2.7432
Ni5	Ni6	2.4687
Ni5	Ni10	2.4489
Ni5	Ni11	2.4524
Ni6	Ni11	2.4365
Ni6	Ni14	2.4079
Ni6	Ni17	2.3982
Ni7	Ni8	2.5211
Ni7	Ni9	2.3621
Ni7	Ni12	2.6467
Ni8	Ni9	2.5781
Ni8	Ni13	2.5240
Ni8	Ni15	2.4601
Ni8	Ni16	2.5469
Ni9	Ni11	2.4111
Ni9	Ni12	2.2810
Ni9	Ni16	2.3384
Ni9	Ni17	2.5511
Ni10	Ni11	2.4213
Ni10	Ni12	2.3966
Ni11	Ni12	2.5120
Ni11	Ni17	2.4003
Ni13	Ni15	2.3658
Ni14	Ni15	2.4707
Ni14	Ni16	3.1821
Ni14	Ni17	2.4908
Ni15	Ni16	2.3857
Ni16	Ni17	2.3608
average bond length		2.515216

$Ni_{18}@(ZnO)_{42}$		
Atom1	Atom2	Length
Ni1	Ni2	2.4231
Ni1	Ni4	2.4421
Ni1	Ni5	2.3613
Ni1	Ni9	2.4542
Ni1	Ni10	2.4164
Ni1	Ni14	2.4111
Ni2	Ni3	2.4155
Ni2	Ni5	2.3989
Ni2	Ni7	2.3287
Ni2	Ni8	2.6860
Ni2	Ni9	2.4770
Ni2	Ni12	2.3988
Ni2	Ni14	2.4089
Ni2	Ni15	2.4769
Ni2	Ni16	2.7001
Ni3	Ni9	2.5749
Ni3	Ni12	2.7023
Ni3	Ni15	2.3288
Ni3	Ni17	2.6256
Ni4	Ni5	2.5544
Ni4	Ni6	2.6931
Ni4	Ni10	2.6299
Ni4	Ni14	2.3781
Ni5	Ni6	2.3986
Ni5	Ni7	2.5467
Ni5	Ni8	2.4695
Ni5	Ni14	2.6775
Ni6	Ni8	2.5446
Ni6	Ni14	3.3736
Ni7	Ni8	2.6059
Ni7	Ni15	2.4821
Ni7	Ni16	2.4238
Ni8	Ni12	2.9406
Ni8	Ni13	2.7344
Ni8	Ni14	2.3516
Ni8	Ni16	2.2803
Ni8	Ni18	3.2858
Ni9	Ni10	2.4612
Ni9	Ni11	2.5043
Ni9	Ni12	2.5365
Ni9	Ni14	2.5092
Ni10	Ni11	2.5849
Ni10	Ni14	2.3680
Ni11	Ni12	2.4717
Ni11	Ni13	2.4793
Ni11	Ni14	2.3723
Ni12	Ni13	2.3763
Ni12	Ni14	2.4163
Ni12	Ni15	3.2854
Ni12	Ni16	2.4587
Ni12	Ni17	2.3411
Ni12	Ni18	2.3933
Ni13	Ni14	2.4815
Ni13	Ni18	2.4266
Ni15	Ni16	2.2799
Ni15	Ni17	2.4779
Ni16	Ni17	2.4288
Ni16	Ni18	2.2980
Ni17	Ni18	2.5837
average bond length		2.524339
